# Region-Specific Information-Theoretic Feature Representation of Wearable Plantar Insole Signals for Parkinson’s Disease Gait Assessment

**DOI:** 10.3390/bios16070391

**Published:** 2026-07-20

**Authors:** Hao Li, Xinyu Zhang, Qikai Wang, Jun Ma

**Affiliations:** Department of Infocommunication Technologies, Belarusian State University of Informatics and Radioelectronics, 220013 Minsk, Belarus

**Keywords:** Parkinson’s disease, gait assessment, plantar insole sensors, wearable sensing, information-theoretic analysis, self-information index

## Abstract

Parkinson’s disease (PD) is associated with gait impairment, bilateral asymmetry, and increased gait variability, highlighting the need for objective and interpretable wearable gait assessment. Plantar insole recordings directly capture foot–ground loading, but their use in PD assessment is often limited by global or low-order descriptors that do not fully represent regional loading organization. This study proposes a region-specific information-theoretic framework for PD gait assessment using wearable plantar-pressure insoles. Bilateral plantar insole signals were reorganized into five anatomical regions: heel, rearfoot, midfoot, forefoot, and toe. Self-information index (SII), Shannon entropy (EN), negentropy (NEG), sample entropy (SEN), and Kullback–Leibler divergence (KL) features were extracted to characterize self-information fluctuation, probabilistic uncertainty, non-Gaussian organization, temporal irregularity, and directional distributional discrepancy in plantar-pressure dynamics. The resulting feature representation was evaluated at gait-cycle, walking-recording, and subject-independent levels using conventional classifiers, ablation analysis, subject-balanced cycle aggregation, and an information-theoretic three-dimensional feature-space rule model (ITFS-RM). KNN achieved an accuracy of 0.9668 at the gait-cycle level, and MLP achieved an accuracy of 0.9344 at the walking-recording level. Under stricter subject-independent evaluation, the accuracy was 0.8475, and subject-balanced-cycle aggregation achieved an accuracy of 0.8655. Region-specific analysis and ablation experiments showed spatially heterogeneous HC–PD differences, with the toe region showing the most consistent contribution. SII, KL, and NEG provided stable discriminative contributions, particularly in toe-related and regional-transition features. ITFS-RM provided explicit feature combinations, value ranges, and spatial rule boundaries for interpretable walking-recording level and subject-grouped separation. These results support region-specific information-theoretic analysis as an interpretable representation of plantar-pressure dynamics for PD gait assessment and emphasize the need for subject-wise validation when repeated walking recordings are available.

## 1. Introduction

Parkinson’s disease (PD) is a progressive neurodegenerative disorder in which gait dysfunction is one of the most disabling motor manifestations. In addition to bradykinesia, rigidity, tremor, and postural instability, patients with PD commonly exhibit shortened stride length, increased gait variability, impaired bilateral coordination, and reduced adaptability during walking [1,2,3,4,5]. Because these abnormalities evolve with disease progression and may fluctuate across clinical and daily-life contexts, quantitative gait assessment is important for objective phenotyping, longitudinal follow-up, and the exploration of candidate digital gait features in PD [2,5,6,7].

The development of wearable sensing technologies has expanded the potential application scenarios of PD gait assessment, enabling objective and repeatable monitoring beyond purely laboratory-based systems [2,5,6,7]. Among different wearable modalities, plantar insole sensors are particularly suitable for PD gait assessment because they directly record foot–ground interactions and plantar loading patterns during walking [8,9,10]. These signals may reveal regional loading abnormalities, bilateral asymmetry, and propulsion-related changes that are not fully captured by global spatiotemporal parameters alone [8,9,10]. Recent studies have further shown that smart insoles can support portable gait-abnormality detection, wearable gait monitoring, and Parkinsonian gait recognition even under reduced plantar-sensor configurations [9,11,12].

Despite these advances, a large portion of existing PD gait research still mainly relies on conventional spatiotemporal parameters such as gait speed, cadence, stride length, stance time, swing time, and their related variability indices [1,2,3,4,7]. These measures have clear clinical value, but they mainly reflect average behavior or low-order statistical characteristics and may be insufficient for capturing the richer dynamic structure embedded in plantar-pressure time series. Insole signals exhibit not only spatial distribution across plantar regions but also temporal organization within gait cycles, reflecting the evolution, fluctuation, and reorganization of loading states during walking. For PD, pathological gait is characterized not only by slower speed or shorter stride length but also by stronger asymmetry, poorer stability, and higher irregularity [4,5]. Related studies have shown that plantar-pressure-based insole analysis can provide informative representations for gait monitoring and motor-function assessment beyond conventional summary descriptions alone [10,13,14]. Therefore, a more informative and interpretable representation of plantar insole signals is needed for Parkinsonian gait assessment.

Information-theoretic analysis provides a suitable framework for modeling plantar-pressure signals because it can characterize uncertainty, irregularity, non-Gaussian organization, and distributional discrepancy beyond mean- or variance-based features. In this study, five complementary information-theoretic features are considered. Shannon entropy is used to characterize the uncertainty of the signal amplitude distribution and reflects the dispersion or concentration of pressure states [15]. The self-information index (SII) describes the relative fluctuation of self-information within regional plantar loading states and provides an additional representation of information-structure variability [13]. Negentropy characterizes the deviation of the signal distribution from a Gaussian reference and captures non-Gaussian organization in plantar-pressure signals [16]. Sample entropy quantifies temporal irregularity in finite time series and is suitable for physiological signals with unstable or weakly repeatable patterns [17]. Kullback–Leibler (KL) divergence quantifies directional distributional discrepancy between plantar loading distributions, including bilateral same-region differences and adjacent-region transitions along the plantar anatomical axis.

A single information measure is usually insufficient to fully characterize the complex organization of plantar-pressure signals. Signals with similar amplitude uncertainty may still differ in temporal regularity, bilateral asymmetry, or heel-to-toe loading transition. Therefore, integrating entropy-based, self-information-based, non-Gaussianity-based, temporal-irregularity-based, and distributional-divergence-based features may provide a more comprehensive representation of Parkinsonian plantar-pressure dynamics. In feature-mining studies based on insole data, sorting-related and information-related features have also been explored as potentially useful ways to characterize differences in plantar-signal organization [13,18].

In addition to feature type, the organization of plantar signals also affects interpretability. Existing plantar-pressure studies often use either whole-foot summary measures or channel-wise representations. Whole-foot aggregation may obscure local abnormalities, whereas channel-wise modeling may introduce considerable noise and increase the difficulty of clinical interpretation. Therefore, this study adopts a region-specific framework in which bilateral insole signals are reorganized into five functionally meaningful regions: heel, rearfoot, midfoot, forefoot, and toe. This design follows the functional transfer process of plantar loading during gait, while reducing dimensional redundancy and improving the interpretability of downstream modeling. Region-level analysis is particularly important for plantar-pressure-based gait assessment because abnormalities in loading distribution, asymmetry, and propulsion may not be uniformly distributed across the whole plantar surface [9,10,11,12]. Studies on balance-related plantar-pressure analysis have also supported the value of region-aware insole representations for identifying subtle differences in motor control [13,19].

Based on these considerations, this study constructs a region-specific information-theoretic framework for gait assessment in Parkinson’s disease. Accordingly, the proposed framework combines three methodological components into a unified analytical pipeline. Bilateral plantar insole signals are first reorganized into five anatomically meaningful regions, which preserves heel-to-toe plantar loading organization while avoiding reliance solely on whole-foot summary features or isolated sensor-channel features. On this regional basis, self-information index (SII), Shannon entropy (EN), negentropy (NEG), sample entropy (SEN), and Kullback–Leibler divergence (KL) features are jointly used to characterize complementary properties of plantar-pressure dynamics, including self-information fluctuation, distributional uncertainty, non-Gaussian organization, temporal irregularity, and directional differences between plantar regions or foot sides. In parallel, the information-theoretic three-dimensional feature-space rule model (ITFS-RM) projects these regional information-theoretic features into explicit three-dimensional rule spaces, enabling HC–PD separation to be described through interpretable feature combinations, value ranges, and spatial decision regions.

The main contributions of this study are as follows.

(1) A region-specific plantar representation is constructed by reorganizing bilateral insole sensor recordings into five anatomical regions: heel, rearfoot, midfoot, forefoot, and toe. This representation preserves heel-to-toe plantar loading organization while reducing the dependence on whole-foot averages or isolated channel-wise features.
(2) A complementary information-theoretic feature set is developed by integrating SII, EN, NEG, SEN, and KL features. These features characterize plantar-pressure dynamics from the perspectives of self-information fluctuation, distributional uncertainty, non-Gaussian organization, temporal irregularity, and directional distributional discrepancy.
(3) An information-theoretic three-dimensional feature-space rule model (ITFS-RM) is proposed to identify class-pure spatial rule regions from regional information-theoretic plantar features. The model provides explicit feature combinations, value ranges, and spatial rule boundaries, thereby offering an interpretable decision framework for walking-recording level and subject-grouped HC–PD separation.
(4) Subject-independent evaluation is conducted by keeping all walking recordings and gait cycles from the same subject within the same data subset. Subject-grouped cross-validation, Leave-One-Subject-Out validation, independent testing, and subject-balanced 50-cycle aggregation are used to distinguish walking-condition-specific separability from generalization to unseen subjects.

The remainder of this paper is organized as follows. Section 2 describes the wearable plantar insole dataset, signal preprocessing, regional plantar reorganization, gait-cycle segmentation, information-theoretic feature extraction, KL-based distributional feature construction, ITFS-RM, subject-independent evaluation, and classification framework. Section 3 presents the experimental results, including region-specific feature characterization, interpretable rule analysis, classification performance, subject-independent generalization evaluation, robustness and computational cost analysis, baseline comparison, and ablation analysis. Section 4 discusses the biomechanical implications, predictive performance, interpretability, comparison with existing studies, limitations, and future work. Section 5 concludes the paper.

## 2. Materials and Methods

### 2.1. Wearable Plantar Insole Dataset and Signal Representation

The publicly available Gait in Parkinson’s Disease database from PhysioNet [20] was used for analysis. The database demographic records include 93 participants with idiopathic Parkinson’s disease (PD) and 73 healthy control (HC) participants. The study included 165 subjects with available walking-recording files, comprising 93 patients with Parkinson’s disease and 72 healthy controls (excluding one HC participant without a corresponding recording file). During data acquisition, participants walked on level ground at a self-selected pace for approximately 2 min. Bilateral plantar insole force signals were recorded at 100 Hz using eight force sensors under each foot. Each available walking-recording file contains 19 columns, including time, eight vertical ground reaction force signals under the left foot, eight vertical ground reaction force signals under the right foot, and the total force under each foot.

In the PhysioNet database, walking recordings are stored as individual experimental text files, and the file names encode the source study, group label, participant identifier, and walking-trial number. According to the database format description, “Co” and “Pt” denote healthy control and PD patient recordings, respectively, whereas the final walking-trial number indicates the specific walking condition or repeated recording. The dataset contained 306 walking-recording files. Each walking-recording file corresponded to one complete walking acquisition rather than to one unique subject. Since some subjects contributed multiple walking recordings under different walking conditions, walking speeds, or task states, a single subject could correspond to multiple walking-recording files.

The available walking-recording suffixes in this study were 01–07 and 10. Suffix 01 denotes usual walking, whereas suffix 10 in the Ga study denotes serial-7 subtraction dual-task walking. Suffixes 02–07 reflect other walking trials under different walking speeds or study-specific experimental protocols. Therefore, analyzing walking-recording files was necessary because it preserved complete walking-recording level gait differences across walking conditions, walking speeds, and task states.

At the same time, subject-level grouped analysis was also necessary. Multiple walking recordings from the same subject share individual-specific gait characteristics. If model partitioning is performed only at the recording-file level, different recordings from the same subject may appear simultaneously in model development and performance evaluation. Therefore, this study adopted two complementary analysis levels. First, walking-recording files were used as walking-recording level samples to evaluate the discriminative ability of region-specific information-theoretic features for HC and PD under different walking speeds and task states. Second, subject identifiers were used as grouping variables, and all walking recordings and corresponding gait cycles from the same subject were kept within the same data subset to evaluate model generalization to unseen subjects. The file-level dataset audit supporting this subject-level grouping is provided as Table S1 in the Supplementary Materials. It summarizes recording identifiers, walking-trial information, available walking speeds, valid gait-cycle counts, inclusion and exclusion status, and data-subset allocation.

Let F∈{l,r} indicate the left and right foot, respectively; let s=1,2,…,8 denote the sensor index; and let $t_{n}$ be the *n*-th sampling instant. The raw plantar insole signal is represented as

(1) $$p_{s,F}\left(t_{n}\right),$$

where $p_{s,F}\left(t_{n}\right)$ denotes the signal acquired from sensor *s* on foot side *F* at time $t_{n}$.

The analytical pipeline consisted of signal preprocessing, plantar regional reorganization, adaptive threshold-based gait cycle segmentation, region-specific information-theoretic feature extraction, Kullback–Leibler divergence-based distributional feature construction, feature matrix generation, walking-condition-specific walking-recording level classification, and subject-independent validation.

**Figure 1:**
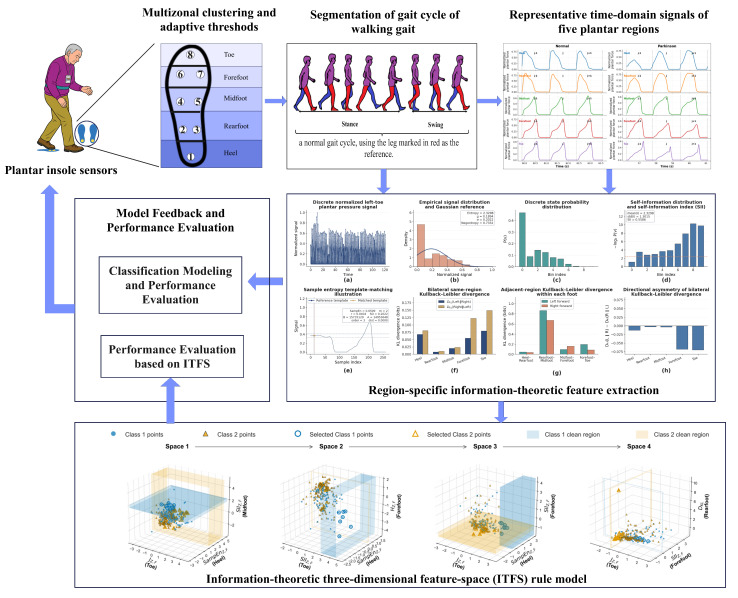
Analytical framework of the proposed region-specific information-theoretic plantar insole analysis for Parkinson’s disease gait assessment. Bilateral plantar insole signals are first reorganized into five anatomical plantar regions, and adaptive thresholding is then used for gait-cycle segmentation. Regional plantar time-domain signals are generated and transformed into region-specific information-theoretic features and KL-based distributional difference features. These features are further used to construct an information-theoretic three-dimensional feature-space (ITFS) rule model for classification modeling, performance evaluation, and model-feedback assessment. (**a**) shows the temporal signal; (**b**) the empirical distribution and Gaussian reference; (**c**) the discrete-state probabilities; (**d**) the self-information distribution and SII; (**e**) sample-entropy template matching; (**f**) bilateral same-region Kullback–Leibler divergence; (**g**) adjacent-region Kullback–Leibler divergence within each foot; and (**h**) directional bilateral Kullback–Leibler asymmetry. Numbers 1–8 directly indicate the positions of the eight plantar sensors.

Figure 1 illustrates the analytical framework of the proposed region-specific information-theoretic plantar insole analysis for Parkinson’s disease gait assessment. The framework integrates plantar region reorganization, gait-cycle segmentation, information-theoretic feature extraction, KL-based distributional modeling, and an information-theoretic three-dimensional feature-space (ITFS) rule model, enabling both walking-condition-specific walking-recording level analysis and subject-independent generalization evaluation.

### 2.2. Plantar Signal Preprocessing, Regional Reorganization, and Gait-Cycle Segmentation

Each sensor channel was smoothed using a centered moving-average filter to reduce local fluctuations. Missing values introduced at signal boundaries were completed by forward and backward filling. Each channel was normalized by its within-record maximum:

(2) $${\tilde{p}}_{s,F}\left(t_{n}\right)=\frac{p_{s,F}\left(t_{n}\right)}{\max_{n}p_{s,F}\left(t_{n}\right)},$$

where ${\tilde{p}}_{s,F}\left(t_{n}\right)$ denotes the normalized signal, and $\max_{n}p_{s,F}\left(t_{n}\right)$ denotes the maximum value of sensor *s* on foot side *F* within the same recording.

To construct a structured plantar representation, the eight sensors on each foot were reorganized into five plantar regions. As illustrated in the multizonal clustering and adaptive thresholds module of Figure 1, the sensor layout was grouped into heel, rearfoot, midfoot, forefoot, and toe regions as part of the overall analytical framework. Let Z∈{H,RF,MF,FF,T} denote heel, rearfoot, midfoot, forefoot, and toe, respectively.

The regional plantar signals were defined as

(3) $$p_{H,F}\left(t_{n}\right)={\tilde{p}}_{1,F}\left(t_{n}\right),$$

(4) $$p_{RF,F}\left(t_{n}\right)=\frac{{\tilde{p}}_{2,F}\left(t_{n}\right)+{\tilde{p}}_{3,F}\left(t_{n}\right)}{2},$$

(5) $$p_{MF,F}\left(t_{n}\right)=\frac{{\tilde{p}}_{4,F}\left(t_{n}\right)+{\tilde{p}}_{5,F}\left(t_{n}\right)}{2},$$

(6) $$p_{FF,F}\left(t_{n}\right)=\frac{{\tilde{p}}_{6,F}\left(t_{n}\right)+{\tilde{p}}_{7,F}\left(t_{n}\right)}{2},$$

(7) $$p_{T,F}\left(t_{n}\right)={\tilde{p}}_{8,F}\left(t_{n}\right),$$

where $p_{Z,F}\left(t_{n}\right)$ denotes the normalized regional plantar signal of region *Z* on foot side *F* at time $t_{n}$.

Adaptive threshold selection was performed separately for each plantar region using iterative two-cluster partitioning of the regional signal sequence. This K-means-like thresholding strategy is consistent with previous smart-insole gait analysis studies that used clustering-based threshold selection for plantar-load state identification and gait segmentation [21,22]. For each plantar region *Z* and foot side *F*, a two-cluster iterative thresholding procedure was applied to the regional pressure signal. At iteration $I_{c}$, each sample was assigned to the cluster whose current mean was closest to its signal value. Let $C_{k_{i},Z,F,I_{c}}$ denote the set of samples assigned to the $k_{i}$-th cluster at iteration $I_{c}$, where $k_{i}$ indicates the cluster with the smaller mean value. This lower-loading cluster was used to estimate the regional threshold, because it represents the baseline or low-pressure state of the plantar region. The threshold was calculated as the mean value of all samples assigned to this lower-loading cluster:

(8) $$Th_{Z,F}=\frac{1}{N_{C_{k_{i},Z,F}}}\sum_{k_{c}=1}^{N_{C_{k_{i},Z,F}}}C_{k_{i},Z,F,I_{c}}\left(k_{c}\right),$$

where $Th_{Z,F}$ is the threshold of region *Z* on foot side *F*, $C_{k_{i},Z,F,I_{c}}\left(k_{c}\right)$ is the $k_{c}$-th sample assigned to the lower-loading cluster, and $N_{C_{k_{i},Z,F}}$ is the number of samples in that cluster.

Gait-cycle boundaries were then determined from upward crossings of the heel-region threshold, following the plantar-threshold event-detection principle used in previous smart-insole-based gait segmentation studies [21,22]. The heel region was used because heel loading provides a stable initial-contact-related event during walking. An upward threshold crossing was detected when

(9) $$p_{H,F}\left(t_{n-1}\right)<Th_{H,F}\leq p_{H,F}\left(t_{n}\right),$$

where $p_{H,F}\left(t_{n}\right)$ is the heel-region pressure signal at time $t_{n}$, and $Th_{H,F}$ denotes the heel-region threshold on foot side *F*.

Two consecutive upward crossings of the heel-region threshold were used to define one gait cycle. Let $t_{GC,start}\left(j,F\right)$ and $t_{GC,end}\left(j,F\right)$ denote the start and end times of the *j*-th gait cycle on foot side *F*, respectively. The duration of this gait cycle was calculated as

(10) $$TD_{GC}\left(j,F\right)=t_{GC,end}\left(j,F\right)-t_{GC,start}\left(j,F\right),$$

where $TD_{GC}\left(j,F\right)$ denotes the duration of the *j*-th gait cycle on foot side *F*. For region *Z*, the corresponding cycle level signal segment is defined as

(11) $$p_{Z,F}\left(TD_{GC}\left(j,F\right)\right)=\left\{p_{Z,F}\left(t_{n}\right)|t_{GC,start}\left(j,F\right)\leq t_{n}<t_{GC,end}\left(j,F\right)\right\},$$

where $p_{Z,F}\left(TD_{GC}\left(j,F\right)\right)$ denotes the regional plantar sequence extracted from the interval $\left[t_{GC,start}\left(j,F\right),\;t_{GC,end}\left(j,F\right)\right)$.

**Figure 2:**
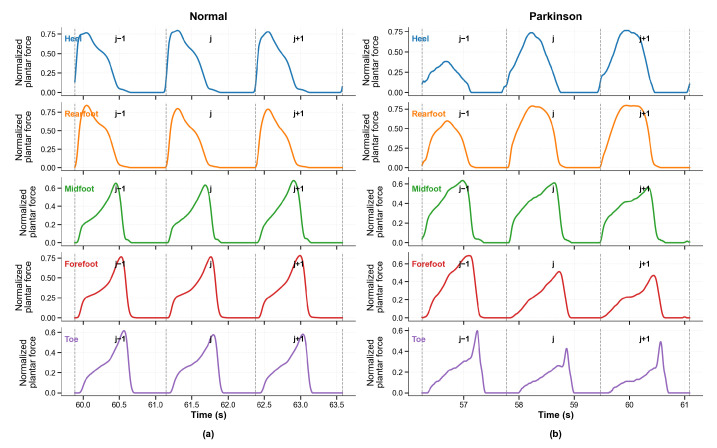
Representative time-domain signals of five plantar regions in a healthy control (**a**) and a patient with Parkinson’s disease (**b**).

Representative time-domain signals of the five plantar regions over three consecutive gait cycles are shown in Figure 2. The three cycles are denoted as j−1, *j*, and j+1, and the vertical dashed lines indicate heel-strike events detected from upward crossings of the heel-region threshold and are shown across all regional signals to illustrate the corresponding gait-cycle boundaries.

### 2.3. Region-Specific Information-Theoretic Feature Extraction

For each valid gait cycle, region-specific information-theoretic descriptors were extracted from the five plantar regions of both feet. For the *j*-th gait cycle, the regional plantar sequence was written as $p_{Z,F}\left(TD_{GC}\left(j,F\right)\right)$, where Z∈{H,RF,MF,FF,T} represents heel, rearfoot, midfoot, forefoot, and toe, respectively, and F∈{l,r} represents the left and right foot. The information-theoretic feature set was defined as

(12) ITF={SII,EN,NEG,SEN,KL},

where ITF is the information-theoretic feature set, SII is the self-information index, EN is Shannon entropy, NEG is negentropy, SEN is sample entropy, and KL is Kullback–Leibler divergence. These descriptors characterize complementary information properties of regional plantar loading, including self-information fluctuation, probabilistic uncertainty, non-Gaussian organization, temporal irregularity, and directional distributional discrepancy.

Shannon entropy was calculated from the discretized cycle level regional signal as

(13) $$EN_{Z,F}\left(j\right)=-\sum_{v=1}^{V}P_{Z,F}^{\left(j\right)}\left(v\right)\log_{2}P_{Z,F}^{\left(j\right)}\left(v\right),$$

where $EN_{Z,F}\left(j\right)$ is the Shannon entropy of region *Z* on foot side *F* in cycle *j*, $P_{Z,F}^{\left(j\right)}\left(v\right)=n_{Z,F}^{\left(j\right)}\left(v\right)/N_{GC}\left(j,F\right)$ is the empirical probability of the *v*-th histogram bin, $n_{Z,F}^{\left(j\right)}\left(v\right)$ is the number of samples falling into the *v*-th bin, $N_{GC}\left(j,F\right)$ is the number of samples in the corresponding gait-cycle sequence, and V=10 is the number of histogram bins. To avoid undefined logarithmic terms when a histogram bin had zero probability, a smoothing constant $\epsilon_{0}=10^{-10}$ was added to each bin probability. The smoothed probability was then renormalized as ${\tilde{P}}_{Z,F}^{\left(j\right)}\left(v\right)=\frac{P_{Z,F}^{\left(j\right)}\left(v\right)+\epsilon_{0}}{\sum_{u=1}^{V}\left(P_{Z,F}^{\left(j\right)}\left(u\right)+\epsilon_{0}\right)}$, so that all bin probabilities were strictly positive and summed to one. This smoothed probability distribution was used in the entropy-, self-information-, negentropy-, and KL-based calculations. Shannon entropy quantifies the uncertainty of the regional plantar loading distribution. A higher value indicates a more dispersed loading-state distribution within the corresponding region and gait cycle.

The self-information index was calculated as

(14) $$SII_{Z,F}\left(j\right)=\frac{\sigma \left(I_{Z,F}^{\left(j\right)}\right)}{{\bar{I}}_{Z,F}^{\left(j\right)}+\epsilon_{0}},$$

where $SII_{Z,F}\left(j\right)$ is the self-information index of region *Z* on foot side *F* in cycle *j*, $I_{Z,F}^{\left(j\right)}\left(v\right)=-\log_{2}P_{Z,F}^{\left(j\right)}\left(v\right)$ is the self-information of state *v*, ${\bar{I}}_{Z,F}^{\left(j\right)}=\sum_{v=1}^{V}P_{Z,F}^{\left(j\right)}\left(v\right)I_{Z,F}^{\left(j\right)}\left(v\right)$ is the probability-weighted mean of self-information, $\sigma \left(I_{Z,F}^{\left(j\right)}\right)=\sqrt{\sum_{v=1}^{V}P_{Z,F}^{\left(j\right)}\left(v\right){\left(I_{Z,F}^{\left(j\right)}\left(v\right)-{\bar{I}}_{Z,F}^{\left(j\right)}\right)}^{2}}$ is the probability-weighted standard deviation of self-information, and $\epsilon_{0}=10^{-10}$ is a numerical constant used to avoid division by zero or unstable ratios when the mean self-information is close to zero. SII measures the relative fluctuation of self-information within a regional plantar sequence. In the proposed framework, it captures within-cycle variability of information content and reflects the stability of plantar loading states in each anatomical region.

Negentropy was used to quantify the departure of the observed regional plantar-loading distribution from a Gaussian reference distribution. The Gaussian reference was not introduced because plantar-pressure signals were assumed to be Gaussian. Instead, it served as a maximum-entropy reference distribution with the same variance as the observed regional signal. Therefore, negentropy provides a compact descriptor of non-Gaussian distributional organization, which may arise from contact–noncontact transitions, stance-phase loading, and asymmetric regional pressure states, consistent with its use as a measure of departure from Gaussianity in signal analysis [16].

Negentropy was calculated as

(15) $$NEG_{Z,F}\left(j\right)=\max\left(h_{Z,F}^{G}\left(j\right)-h_{Z,F}^{obs}\left(j\right),0\right),$$

where $NEG_{Z,F}\left(j\right)$ is the negentropy of region *Z* on foot side *F* in cycle *j*, $h_{Z,F}^{obs}\left(j\right)=H_{Z,F}^{disc}\left(j\right)+\log_{2}\left(\Delta b+\epsilon_{0}\right)$ is the histogram-based entropy approximation of the observed regional signal, $H_{Z,F}^{disc}\left(j\right)=-\sum_{v=1}^{V}P_{Z,F}^{\left(j\right)}\left(v\right)\log_{2}P_{Z,F}^{\left(j\right)}\left(v\right)$ is the discrete histogram entropy of the observed regional signal, Δb is the average histogram bin width, $h_{Z,F}^{G}\left(j\right)=\frac{1}{2}\log_{2}\left(2\pi e\sigma_{Z,F}^{2}\left(j\right)\right)$ is the entropy of a Gaussian reference distribution with the same variance, and $\sigma_{Z,F}^{2}\left(j\right)$ is the variance of the observed regional plantar sequence. Here, $\epsilon_{0}=10^{-10}$ was used to avoid undefined logarithmic values when the histogram bin width was extremely small. If the regional signal had zero or near-zero variance, $NEG_{Z,F}\left(j\right)$ was set to zero.

Sample entropy was calculated as

(16) $$SEN_{Z,F}\left(j\right)=-\ln\left(\frac{A_{Z,F}\left(m+1,r\right)+\epsilon_{0}}{B_{Z,F}\left(m,r\right)+\epsilon_{0}}\right),$$

where $SEN_{Z,F}\left(j\right)$ is the sample entropy of region *Z* on foot side *F* in cycle *j*, m=2 is the embedding dimension, $r=0.2\sigma_{x}$ is the tolerance threshold, $\sigma_{x}$ is the standard deviation of the corresponding regional plantar sequence, $A_{Z,F}\left(m+1,r\right)$ is the number of matched vector pairs at embedding dimension m+1 within tolerance *r*, $B_{Z,F}\left(m,r\right)$ is the number of matched vector pairs at embedding dimension *m* within tolerance *r*, and $\epsilon_{0}=10^{-10}$ is a numerical constant used to avoid undefined logarithmic values when no matched vector pairs are found in either the numerator or denominator. Sample entropy quantifies the temporal irregularity of the regional plantar signal. In this framework, it was used to characterize the regularity and complexity of plantar loading fluctuations within each gait cycle.

Kullback–Leibler divergence was used to quantify directional distributional differences between regional plantar signals. For two regional signal sequences *x* and *y*, the KL divergence from $P_{x}$ to $P_{y}$ was calculated as

(17) $$D_{KL}\left(P_{x}\|P_{y}\right)=\sum_{v=1}^{V}P_{x}\left(v\right)\log_{2}\frac{P_{x}\left(v\right)}{P_{y}\left(v\right)},$$

where $D_{KL}\left(P_{x}\|P_{y}\right)$ is the directional distributional discrepancy from signal *x* to signal *y*, $P_{x}\left(v\right)$ and $P_{y}\left(v\right)$ are the empirical probabilities of the *v*-th histogram bin estimated from signals *x* and *y* using a common bin partition constructed from their combined value range, V=10 is the number of histogram bins, and the logarithm base 2 gives the divergence in bits. A smoothing constant $\epsilon =10^{-10}$ was added to each bin probability followed by probability renormalization. In this framework, KL features were used to describe plantar-load redistribution between homologous bilateral regions and distributional transitions between adjacent regions within the same foot.

For inter-foot same-region comparison, the bidirectional KL features were calculated as

(18) $$KL_{Z}^{l\to r}\left(j\right)=D_{KL}\left(P_{Z,l}^{\left(j\right)}\|P_{Z,r}^{\left(j\right)}\right),$$

(19) $$KL_{Z}^{r\to l}\left(j\right)=D_{KL}\left(P_{Z,r}^{\left(j\right)}\|P_{Z,l}^{\left(j\right)}\right),$$

(20) $$\Delta KL_{Z}^{lr}\left(j\right)=KL_{Z}^{l\to r}\left(j\right)-KL_{Z}^{r\to l}\left(j\right),$$

where $KL_{Z}^{l\to r}\left(j\right)$ and $KL_{Z}^{r\to l}\left(j\right)$ are the left-to-right and right-to-left KL divergences of region *Z* in cycle *j*, respectively; $P_{Z,l}^{\left(j\right)}$ and $P_{Z,r}^{\left(j\right)}$ are the empirical probability distributions of the left and right regional plantar sequences; and $\Delta KL_{Z}^{lr}\left(j\right)$ is the signed directional KL difference. These features quantify bilateral distributional asymmetry in homologous plantar regions.

For intra-foot adjacent-region comparison, the bidirectional KL features were calculated as

(21) $$KL_{F}^{Z_{a}\to Z_{b}}\left(j\right)=D_{KL}\left(P_{Z_{a},F}^{\left(j\right)}\|P_{Z_{b},F}^{\left(j\right)}\right),$$

(22) $$KL_{F}^{Z_{b}\to Z_{a}}\left(j\right)=D_{KL}\left(P_{Z_{b},F}^{\left(j\right)}\|P_{Z_{a},F}^{\left(j\right)}\right),$$

(23) $$\Delta KL_{F}^{Z_{a}Z_{b}}\left(j\right)=KL_{F}^{Z_{a}\to Z_{b}}\left(j\right)-KL_{F}^{Z_{b}\to Z_{a}}\left(j\right),$$

where $\left(Z_{a},Z_{b}\right)\in \left\{\left(H,RF\right),\left(RF,MF\right),\left(MF,FF\right),\left(FF,T\right)\right\}$ is an adjacent plantar-region pair, $KL_{F}^{Z_{a}\to Z_{b}}\left(j\right)$ and $KL_{F}^{Z_{b}\to Z_{a}}\left(j\right)$ are the bidirectional KL divergences between two adjacent regions on foot side *F*, $P_{Z_{a},F}^{\left(j\right)}$ and $P_{Z_{b},F}^{\left(j\right)}$ are the corresponding empirical probability distributions estimated using a common bin partition, and $\Delta KL_{F}^{Z_{a}Z_{b}}\left(j\right)$ is the signed directional difference. These adjacent-region KL features characterize the distributional transition of plantar loading along the heel-to-toe anatomical axis.

For the complete information-theoretic feature set ITF={SII,EN,NEG,SEN,KL}, bilateral or directional difference features were constructed according to the mathematical form of each descriptor. For scalar descriptors in $ITF_{scalar}=\left\{SII,EN,NEG,SEN\right\}$, the inter-foot difference was defined as

(24) $$\Delta Q_{Z,lr}\left(j\right)=Q_{Z,l}\left(j\right)-Q_{Z,r}\left(j\right),$$

where $Q\in ITF_{scalar}$, $\Delta Q_{Z,lr}\left(j\right)$ is the signed left–right difference of descriptor *Q* in region *Z* for cycle *j*, and $Q_{Z,l}\left(j\right)$ and $Q_{Z,r}\left(j\right)$ are the corresponding scalar descriptor values of the left and right foot. For KL descriptors, the directional difference was defined by $\Delta KL_{Z}^{lr}\left(j\right)$ and $\Delta KL_{F}^{Z_{a}Z_{b}}\left(j\right)$, as given above. This formulation preserves the directionality of KL divergence while maintaining a unified information-theoretic feature set.

**Figure 3:**
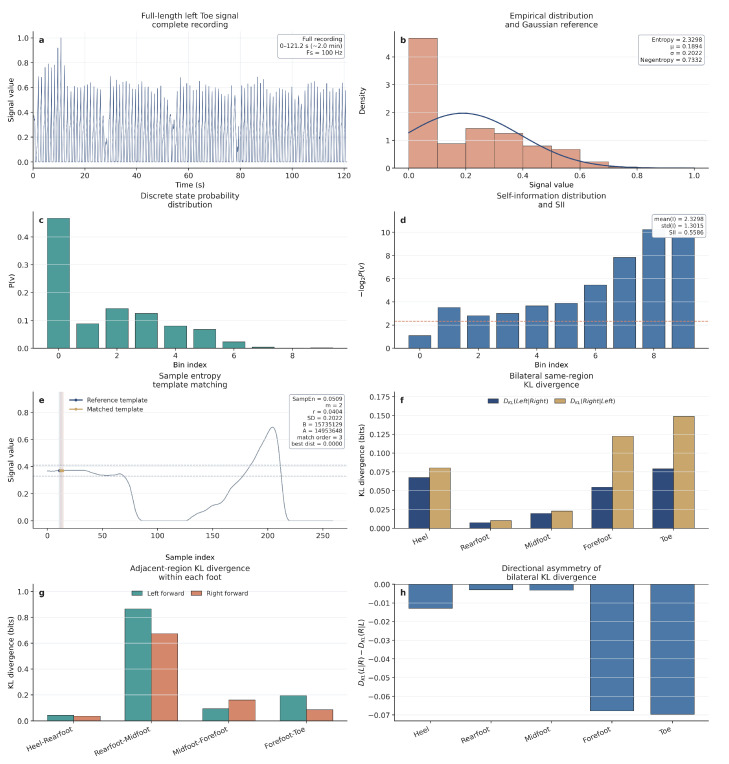
Illustrative visualization of region-specific plantar insole signal descriptors. A representative normalized left-toe plantar pressure signal is shown with derived information-theoretic representations: (**a**) temporal signal basis; (**b**) empirical distribution and Gaussian reference; (**c**) discrete state probabilities; (**d**) self-information distribution and SII, where the red dashed line indicates the probability-weighted mean self-information; (**e**) sample-entropy template matching, where the blue and red shaded areas indicate the reference-template and matched-template intervals, respectively, and the gray dashed horizontal lines indicate an illustrative tolerance range centered on the mean of the reference template, with bounds defined as the reference-template mean ±r; (**f**) bilateral same-region Kullback–Leibler divergence; (**g**) adjacent-region Kullback–Leibler divergence within each foot; and (**h**) directional asymmetry of bilateral Kullback–Leibler divergence.

A representative normalized left-toe plantar insole signal was selected to illustrate the construction of the information-theoretic feature set. Figure 3a–e show the temporal signal basis, empirical distribution with Gaussian reference, discrete state probabilities, self-information distribution with SII, and sample-entropy template matching, respectively. Figure 3f–h show KL-based distributional descriptors, including bilateral same-region divergence, intra-foot adjacent-region divergence, and directional left–right asymmetry. Collectively, Figure 3 summarizes how a regional plantar signal is transformed into features reflecting temporal variation, probabilistic uncertainty, self-information fluctuation, non-Gaussian organization, temporal irregularity, and directional distributional divergence. The visualization serves as a methodological illustration of feature construction and is not used as an input to statistical testing or classification.

### 2.4. Feature Matrix Construction

Information-theoretic features were organized at both gait-cycle and walking-recording levels, with subject identifiers retained for subject-grouped validation. For gait-cycle level analysis, each valid gait cycle was represented by one feature vector, yielding

(25) $$X_{cycle}\in R^{M\times P},$$

where *M* is the number of valid gait cycles, and *P* is the number of gait-cycle level features.

For walking-condition-specific walking-recording level analysis, each walking-recording file was treated as one complete walking-recording level sample rather than as one unique subject. Unlike gait-cycle level analysis, walking-recording level features were extracted directly from the complete recording signal without gait-cycle segmentation. Specifically, after preprocessing and five-region reorganization, SII, EN, NEG, SEN, and KL-related features were calculated from the full-length regional plantar signals of each recording file. Therefore, each recording file generated one walking-recording level feature vector, yielding

(26) $$X_{rec}\in R^{N_{r}\times P_{r}},$$

where $N_{r}$ is the number of walking-recording files used as walking-recording level samples, and $P_{r}$ is the number of walking-recording level features.

For subject-independent validation, the subject identifier was used as the grouping variable. All walking-recording files and gait-cycle feature vectors derived from the same subject were assigned to the same data subset.

The constructed feature set included SII, EN, NEG, SEN, KL, bilateral scalar-difference features, inter-foot same-region KL features, intra-foot adjacent-region KL features, and KL directional-difference features. The class label of sample *j* was defined as

(27) y(j)∈{0,1},

where y(j)=1 represents PD, and y(j)=0 represents HC.

The walking-recording level matrix $X_{rec}$ was subsequently used for the proposed information-theoretic three-dimensional feature-space rule model and walking-recording level supervised classification, whereas $X_{cycle}$ was used for gait-cycle level classification. Subject identifiers were retained in both matrices to support subject-grouped cross-validation, LOSO validation, and subject-independent performance evaluation.

### 2.5. Information-Theoretic Three-Dimensional Feature-Space Rule Model

To provide an interpretable walking-recording level decision framework with subject-grouped evaluation, an information-theoretic three-dimensional feature-space rule model (ITFS-RM) was proposed. Within this framework, ITFS-RM uses walking-recording level regional information-theoretic features to construct explicit three-dimensional feature spaces, identify class-pure HC and PD rule regions, and describe the spatial separation between groups using interpretable decision rules. In contrast to conventional classifiers with limited intrinsic interpretability, ITFS-RM retains the selected feature combination, threshold interval, and rule region, thereby providing a direct interpretation of the information-theoretic structure underlying the decision process.

The main rationale of ITFS-RM is that a discriminative information structure may emerge from the joint spatial distribution of multiple information-theoretic features. SII reflects self-information fluctuation, EN reflects probabilistic uncertainty, NEG reflects non-Gaussian organization, SEN reflects temporal irregularity, and KL reflects directional distributional discrepancy. Therefore, each three-dimensional feature space represents a local information-coordinate system for characterizing complementary information properties of plantar gait signals.

**Figure 4:**
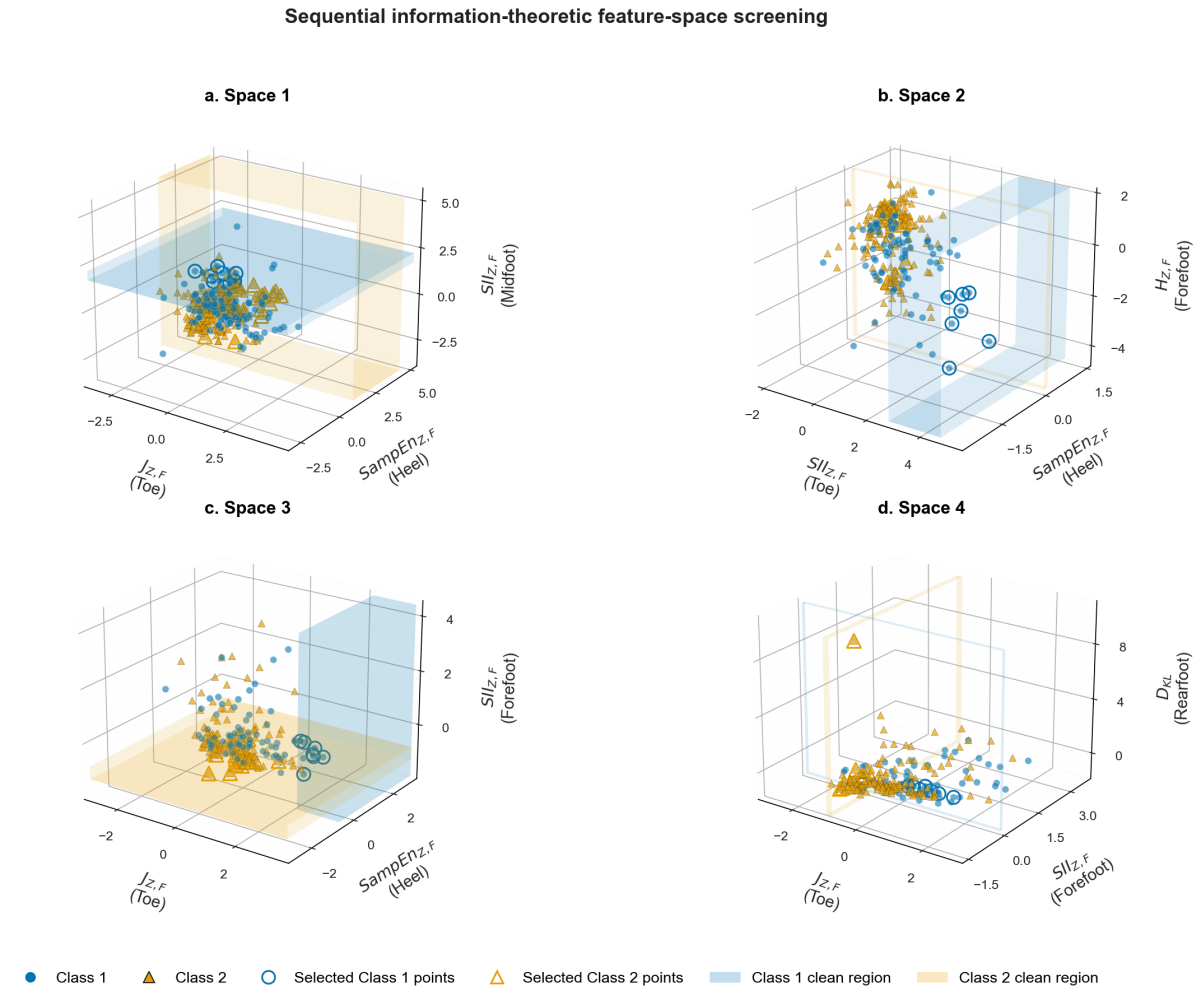
Schematic illustration of the proposed information-theoretic three-dimensional feature-space rule model. Sequential three-dimensional feature spaces are constructed from walking-recording level information-theoretic features. In each space, class-pure rule regions are identified for HC and PD, and recordings covered by these regions are progressively assigned using interpretable spatial rules.

The overall structure of ITFS-RM is shown in Figure 4. Sequential three-dimensional information-theoretic feature spaces are constructed from walking-recording level features. Within each space, class-pure rule regions are identified for HC and PD, and recordings covered by these regions are assigned according to interpretable spatial rules.

Let the walking-recording level feature matrix be

(28) $$X_{rec}={\left[x_{i1},x_{i2},\ldots ,x_{iP_{r}}\right]}_{i=1}^{N_{r}},$$

where $N_{r}$ is the number of walking-recording samples, $P_{r}$ is the number of walking-recording level features, and $x_{ip}$ is the value of the *p*-th feature for recording *i*. The information-theoretic feature set used in ITFS-RM is defined as

(29) $$F_{ITF}=\left\{f_{1},f_{2},\ldots ,f_{P_{r}}\right\},$$

where $F_{ITF}$ includes SII, EN, NEG, SEN, and KL features.

All candidate three-dimensional feature spaces were generated from three-feature combinations:

(30) $$S=\left\{S_{k}=\left(f_{a},f_{b},f_{c}\right)|f_{a},f_{b},f_{c}\in F_{ITF},\;a<b<c\right\},$$

where $S_{k}$ is the *k*-th three-dimensional information-theoretic feature space. For recording *i*, the coordinate vector in $S_{k}$ is

(31) $$z_{i}^{\left(k\right)}={\left[x_{i}\left(f_{a}\right),x_{i}\left(f_{b}\right),x_{i}\left(f_{c}\right)\right]}^{T},$$

where $x_{i}\left(f_{a}\right)$, $x_{i}\left(f_{b}\right)$, and $x_{i}\left(f_{c}\right)$ are the values of the three selected features.

The subject identifier was retained as a grouping variable. During subject-grouped training, validation, and testing, all walking recordings from the same subject were kept within the same data subset. Thus, ITFS-RM rules were learned from training recordings only and were evaluated on recordings from unseen subjects when subject-independent validation was performed.

To improve the representativeness of the initial feature spaces, candidate spaces were ordered using a balanced strategy across plantar regions and information-theoretic feature categories. The plantar regions included heel, rearfoot, midfoot, forefoot, and toe, and the feature categories included SII, EN, NEG, SEN, and KL. This strategy reduced over-representation of a single anatomical region or a single information-theoretic feature category in the initial spaces.

For each space $S_{k}=\left(f_{a},f_{b},f_{c}\right)$, class-pure rule regions were searched separately for HC and PD. Two types of interpretable rules were considered. The first was a single-axis interval rule:

(32) $$R_{c}^{axis}\left(f_{a}\right)=\left\{i|\alpha_{c}\leq x_{i}\left(f_{a}\right)\leq \beta_{c}\right\},$$

where $R_{c}^{axis}\left(f_{a}\right)$ is the interval rule region for class c∈{HC,PD}, and $\alpha_{c}$ and $\beta_{c}$ are the lower and upper bounds of the interval.

The second was a two-dimensional threshold-combination rule. For class *c*, candidate thresholds were first generated from the empirical percentiles of the target-class samples:

(33) $$T_{a}^{\left(c\right)}=\left\{{\mathrm{Percentile}}_{q}\left(\{x_{i}\left(f_{a}\right)|y_{i}=c\}\right)|q\in Q\right\},$$

(34) $$T_{b}^{\left(c\right)}=\left\{{\mathrm{Percentile}}_{q}\left(\{x_{i}\left(f_{b}\right)|y_{i}=c\}\right)|q\in Q\right\},$$

where $T_{a}^{\left(c\right)}$ and $T_{b}^{\left(c\right)}$ are the candidate threshold sets for features $f_{a}$ and $f_{b}$, respectively, and Q={0,10,20,…,100} is the percentile search grid.

For any $\tau_{a}\in T_{a}^{\left(c\right)}$, $\tau_{b}\in T_{b}^{\left(c\right)}$, and direction parameters $\delta_{a},\delta_{b}\in \left\{-1,1\right\}$, the two-dimensional threshold-combination rule was defined as

(35) $$R_{c}^{quad}\left(f_{a},f_{b};\tau_{a},\tau_{b},\delta_{a},\delta_{b}\right)=\left\{i|\delta_{a}\left[x_{i}\left(f_{a}\right)-\tau_{a}\right]\geq 0,\;\delta_{b}\left[x_{i}\left(f_{b}\right)-\tau_{b}\right]\geq 0\right\},$$

where $\tau_{a}$ and $\tau_{b}$ are the selected feature thresholds. The direction parameter $\delta_{a}=1$ corresponds to $x_{i}\left(f_{a}\right)\geq \tau_{a}$, whereas $\delta_{a}=-1$ corresponds to $x_{i}\left(f_{a}\right)\leq \tau_{a}$. The same definition applies to $\delta_{b}$ for feature $f_{b}$.

A candidate rule region was retained only when it satisfied both the minimum coverage and class-purity conditions:

(36) $$|R_{c}|\geq n_{\min},$$

(37) $$y_{i}=c,\;\forall i\in R_{c},$$

where $|R_{c}|$ is the number of covered recordings, $n_{\min}$ is the minimum coverage threshold, and $y_{i}$ is the true class label of recording *i*. In this study, $n_{\min}=2$.

For each class *c*, the retained rule in the *k*-th feature space was selected by maximizing the coverage among all valid class-pure candidate regions:

(38) $$R_{c}^{\left(k\right)}=\arg\max_{R\in G_{c}^{\left(k\right)}}\left|R\right|,$$

where $G_{c}^{\left(k\right)}$ is the set of candidate rule regions satisfying the coverage and purity conditions in $S_{k}$. Thus, the final retained rule may be either a single-axis interval rule or a two-dimensional threshold-combination rule, depending on which valid rule covers the largest number of target-class recordings.

The rule mapping was performed sequentially across the ordered feature spaces. Let $U_{k}$ be the set of unresolved recordings before stage *k*. After applying the retained HC and PD rule regions, the unresolved set was updated as

(39) $$U_{k+1}=U_{k}\setminus \left(R_{HC}^{\left(k\right)}\cup R_{PD}^{\left(k\right)}\right),$$

where $R_{HC}^{\left(k\right)}$ and $R_{PD}^{\left(k\right)}$ are the retained rule regions for HC and PD in the *k*-th space. Recordings covered by $R_{HC}^{\left(k\right)}$ were assigned to HC, recordings covered by $R_{PD}^{\left(k\right)}$ were assigned to PD, and uncovered recordings remained unresolved. If a recording was covered by both class rules, it was not assigned at that stage to avoid rule conflict.

The full recording set was defined as $\Omega =\{1,2,\ldots ,N_{r}\}$. The unresolved recording set after stage *k* was denoted as $U_{k+1}$, and the assigned recording set after stage *k* was defined as

(40) $$A_{k}=\left\{i\in \Omega |i\notin U_{k+1}\right\}.$$

The cumulative coverage after stage *k* was defined as

(41) $$Coverage_{k}=\frac{|A_{k}|}{N_{r}}=\frac{N_{r}-\left|U_{k+1}\right|}{N_{r}},$$

where $Coverage_{k}$ represents the proportion of recordings assigned by the rule model after stage *k*, $A_{k}$ is the assigned recording set, $U_{k+1}$ is the unresolved recording set, and $N_{r}$ is the total number of recordings.

Because unresolved recordings were not assigned predicted labels by the rule model, classification accuracy was calculated only for the assigned recordings. The assigned-sample accuracy after stage *k* was defined as

(42) $$Acc_{assigned,k}=\frac{\sum_{i\in A_{k}}I\left({\hat{y}}_{i}^{\left(k\right)}=y_{i}\right)}{|A_{k}|},$$

where $Acc_{assigned,k}$ denotes the accuracy among recordings assigned by the rule model after stage *k*, ${\hat{y}}_{i}^{\left(k\right)}$ is the rule-assigned label of recording *i*, $y_{i}$ is the true class label, and I(·) is the indicator function. In the ITFS-RM analysis, overall ACC refers to the accuracy calculated among the samples assigned by the rule model at each stage, corresponding to $Acc_{assigned,k}$.

To jointly describe rule applicability and rule reliability, $Coverage_{k}$ and $Acc_{assigned,k}$ were reported together at each stage. The sequential rule mapping was terminated at stage *k* when any of the following stopping criteria was satisfied:

(43) $$\left\{\begin{array}{cc} U_{k+1}=⌀, & \mathrm{all}\;\mathrm{recordings}\;\mathrm{were}\;\mathrm{assigned},\\ Acc_{assigned,k}\geq Acc_{target}, & \mathrm{the}\;\mathrm{target}\;\mathrm{assigned}-\mathrm{sample}\;\mathrm{accuracy}\;\mathrm{was}\;\mathrm{reached},\\ |Acc_{assigned,k}-Acc_{assigned,k-1}|\leq \varepsilon , & \mathrm{the}\;\mathrm{assigned}-\mathrm{sample}\;\mathrm{accuracy}\;\mathrm{became}\;\mathrm{stable}. \end{array}\right.$$

where $Acc_{target}$ is the predefined target accuracy for assigned samples, and ε is the numerical tolerance.

ITFS-RM provides an interpretable spatial representation of walking-recording level information-theoretic features. Rather than evaluating each feature independently, the model emphasizes the joint structure among complementary information properties. A class-pure region in a three-dimensional feature space indicates that the corresponding feature combination forms a locally discriminative information structure. Therefore, ITFS-RM identifies both the feature-space configuration and the explicit rule region contributing to HC–PD separation.

In the subject-independent evaluation, ITFS-RM outputs were also aggregated at the subject level by combining the rule-assigned results of all recordings belonging to the same subject. ITFS-RM was used as an interpretable spatial rule model before supervised classification. Its outputs included selected three-dimensional feature spaces, class-pure rule regions, cumulative coverage, assigned-sample accuracy, and subject-aggregated performance when subject grouping was applied.

### 2.6. Supervised Classification Framework and Performance Evaluation

Supervised classification was evaluated at both gait-cycle and walking-recording levels, with subject-independent validation further performed using subject identifiers as grouping variables. At the gait-cycle level, each valid gait cycle was represented as one sample, whereas at the recording level, each walking-recording file was represented by one complete walking-recording level feature vector. The same conventional classifier types and parameter settings were used to ensure comparability across gait-cycle and walking-recording level analyses.

Before model fitting, all features were standardized using Z-score normalization. The normalization parameters were estimated from the training set only and then applied to the corresponding testing and validation sets. The standardized feature value was calculated as

(44) $$f_{q}^{*}\left(j\right)=\frac{f_{q}\left(j\right)-\mu_{q}}{\sigma_{q}},$$

where $f_{q}\left(j\right)$ is the original value of the *q*-th feature for sample *j*, $f_{q}^{*}\left(j\right)$ is the standardized feature value, and $\mu_{q}$ and $\sigma_{q}$ are the mean and standard deviation of the *q*-th feature estimated from the training set.

Eight supervised classifiers were evaluated, including DecisionTree, LogisticRegression, K-Nearest Neighbors, RandomForest, GradientBoosting, Multi-Layer Perceptron, Support Vector Machine, and AdaBoost. For reproducibility, random_state=42 was used for stochastic classifiers and data partitioning. DecisionTree, LogisticRegression, RandomForest, and Support Vector Machine were configured with class_weight=balanced. LogisticRegression and Multi-Layer Perceptron were configured with max_iter=500. RandomForest was configured with n_estimators=100, and Support Vector Machine was configured with probability estimates enabled. The K-Nearest Neighbors classifier was configured with n_neighbors=5, weights=uniform, algorithm=auto, leaf_size=30, p=2, and metric=minkowski.

For the walking-condition-specific gait-cycle and walking-recording level analyses, samples were divided into training, testing, and validation subsets at a ratio of 7:2:1. Specifically, the available samples were first divided into a training subset accounting for 70% of the data and a temporary subset accounting for 30% of the data. The temporary subset was then further divided into testing and validation subsets, resulting in final proportions of 70%, 20%, and 10% for training, testing, and validation, respectively. Stratified sampling was preferentially used to preserve the class distribution between the Parkinson’s disease and healthy control groups; when stratification was not feasible, random splitting was used instead.

For gait-cycle level analysis, the results were interpreted as discriminative performance for abnormal gait at the local cycle level, whereas in the walking-recording level results, each walking-recording file was represented by one feature vector and was interpreted as discriminative performance for abnormal gait at the complete walking-recording level.

In addition to the walking-condition-specific holdout evaluation, subject-independent validation was performed by using subject identifiers as grouping variables. In this setting, all walking-recording files and all gait cycles derived from the same subject were retained within the same data subset. This design was used to evaluate whether the learned feature representation generalized to subjects not used during model development. Subject-grouped 5-fold cross-validation and leave-one-subject-out validation were further performed. The cross-validation was configured with n_splits=5, shuffle=True, and random_state=42. This strategy preserved class proportions while ensuring that samples from the same subject did not appear simultaneously in the training and testing folds. Leave-one-subject-out validation was implemented using leave one group out, where one subject and all corresponding samples, or one walking-recording file and all corresponding samples, were held out as the test set in each round, while the remaining data were used for model training.

Classification performance was assessed using accuracy, precision, recall, F1-score, and AUC:

(45) $$Accuracy=\frac{TP+TN}{TP+TN+FP+FN},$$

(46) $$Precision=\frac{TP}{TP+FP},$$

(47) $$Recall=\frac{TP}{TP+FN},$$

(48) $$F1=\frac{2\times Precision\times Recall}{Precision+Recall},$$

where TP, TN, FP, and FN denote true positives, true negatives, false positives, and false negatives, respectively. AUC was calculated from the ROC curve to evaluate the overall discriminative ability of each classifier across different decision thresholds. For 5-fold cross-validation and leave-one-subject-out validation, classification performance was summarized using the mean and 95% confidence interval:

(49) $$\bar{s}\pm 1.96\frac{\sigma_{s}}{\sqrt{K}},$$

where $\bar{s}$ is the mean score across evaluation rounds, $\sigma_{s}$ is the sample standard deviation of the scores, and *K* is the number of evaluation rounds. This interval was used to describe the uncertainty of the mean validation performance across cross-validation or LOSO rounds. Mean ± standard deviation describes the dispersion of fold-wise or subject-wise scores, whereas the 95% confidence interval estimates the uncertainty of the average performance. Therefore, Equation (49) was used for compact reporting of mean model performance and its uncertainty across repeated validation rounds.

Ablation analysis was performed to evaluate the contributions of different plantar regions and information-theoretic descriptors to classification performance. In the regional ablation analysis, features corresponding to one plantar region were removed at a time, including heel, rearfoot, midfoot, forefoot, and toe. In the descriptor-level ablation analysis, all features belonging to one information-theoretic descriptor were removed at a time, including SII, Shannon entropy, negentropy, sample entropy, and Kullback–Leibler divergence. After each ablation, the classifiers were retrained using the same parameter settings as in the complete-feature experiment, and the change in testing performance was used to quantify the relative contribution of the corresponding region or information-theoretic feature.

### 2.7. Deep Learning and Plantar Signal Baseline Comparison

To further evaluate the discriminative effectiveness of the proposed region-specific information-theoretic feature representation, deep learning baseline models were constructed based on both the information-theoretic feature representation and plantar insole sensor signals as auxiliary baseline comparisons.

For the feature-level deep learning comparison, each standardized information-theoretic feature vector was reshaped into a one-dimensional input sequence with a size of $n_{\mathrm{features}}\times 1$. The evaluated deep learning models included CNN, LSTM, RNN, BiLSTM, CNN–LSTM, and CNN–BiLSTM. For the signal-level baseline comparison, the preprocessed plantar insole sensor signals were directly fed into the deep learning models to evaluate the classification performance of end-to-end signal learning relative to the proposed information-theoretic feature representation.

All deep learning models were trained using the Adam optimizer with a learning rate of $1\times 10^{-3}$, and binary cross-entropy was used as the loss function. The maximum number of training epochs was set to 200, and the batch size was set to 16. The hidden dimensions of the recurrent and fully connected layers were both set to 64, and the dropout rate was set to 0.30. For models containing CNN components, the number of convolutional filters, kernel size, and max-pooling size were set to 64, 3, and 2, respectively.

The deep learning baseline models were compared under the same evaluation levels as the conventional classifiers, including gait-cycle level, walking-recording level, and subject-independent subject-aggregated evaluations.

### 2.8. Subject-Independent Model Development and Generalization Evaluation

Based on the walking-condition-specific walking-recording level analysis, this study further conducted subject-independent evaluation by using the subject identifier as the grouping variable to assess model generalization to unseen subjects.

For each subject, all walking-recording files and all valid gait cycles extracted from these recordings were kept within the same data subset. Therefore, data partitioning in training, validation, and testing was organized at the subject level rather than at the individual recording-file or gait-cycle level.

Within the development subjects, model training, hyperparameter selection, and threshold determination were performed without using the independent test subjects. Subject-grouped five-fold cross-validation and leave-one-subject-out validation were used to assess model stability and unseen-subject generalization.

At the gait-cycle level, a subject-balanced k-cycle evidence aggregation strategy was used. In each repeated experiment, k = 50 cycles were sampled from each subject-specific gait-cycle pool. Cycle level prediction scores from the same subject were averaged to obtain the subject-level aggregated score, reducing the influence of unequal gait-cycle counts across subjects while preserving subject-level data partitioning.

For ITFS-RM, the feature spaces, cascade rules, explicit thresholds, and decision boundaries were determined only from the training subjects and then fixed before being applied to the independent test cohort. Performance was reported at both recording and subject-aggregated levels.

### 2.9. Statistical Analysis

Statistical analysis was performed to evaluate group differences in region-specific information-theoretic features between the HC and PD groups. Considering that the extracted information-theoretic features may not follow a normal distribution, between-group comparisons were performed using the Mann–Whitney U test. For multiple comparisons across plantar regions and information-theoretic descriptors, false discovery rate correction was applied. Statistical significance was defined as adjusted p≤0.05. To reduce the influence of repeated gait-cycle samples from the same walking-recording file, statistical comparisons of feature distributions were performed at the walking-recording level. For gait-cycle level features, the median value across valid gait cycles within each walking-recording file was used as the representative value for statistical testing. Only plantar region–descriptor combinations showing statistically significant HC–PD differences after FDR correction were highlighted in the region-specific feature characterization results.

## 3. Results

### 3.1. Region-Specific Information-Theoretic Feature Characterization

**Figure 5:**
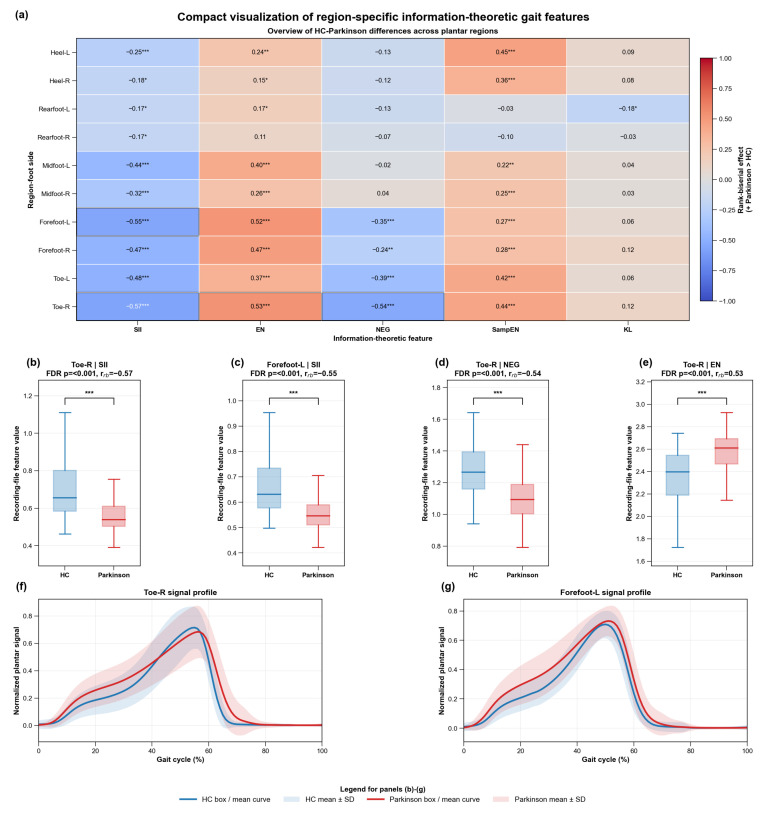
Walking-recording level region-specific information-theoretic differences between HC and PD. (**a**) Heatmap of rank-biserial effect sizes across plantar region–foot-side combinations and information-theoretic features; positive values indicate higher values in PD, and negative values indicate higher values in HC. (**b**–**e**) Distributions of representative discriminative features: Toe-R SII, Forefoot-L SII, Toe-R NEG, and Toe-R EN. (**f**,**g**) Representative normalized plantar signal profiles shown as group mean curves with standard-deviation envelopes. Blue represents HC, and red represents PD. * adjusted p≤0.05, ** adjusted p≤0.01, *** adjusted p≤0.001.

Figure 5 shows the walking-recording level region-specific information-theoretic feature differences between the HC and PD groups. All plantar region–foot-side combinations are summarized in the overview heatmap, and statistically significant group differences are indicated after FDR correction. The results indicate that PD-related differences were not uniformly distributed across the whole plantar surface but were mainly observed in the midfoot, forefoot, and toe regions, with additional feature-specific differences in the heel region.

In the heel region, group differences were mainly reflected in SII, EN, and SEN, whereas NEG and KL showed weaker differences. In the rearfoot region, although the average pressure trajectories were relatively similar between groups, the feature-level differences were comparatively limited and were mainly observed in SII, EN, and KL for specific foot sides. In the midfoot region, group differences were mainly reflected in feature distribution and cycle stability, particularly in SII and entropy-related descriptors. The forefoot region also showed clear group differences, especially for SII and EN, indicating that PD-related changes were not restricted to the posterior plantar regions.

The toe region showed the most prominent group differences. Compared with HC, the PD group exhibited distinct walking-recording level feature shifts and significant differences across multiple features, including SII, EN, NEG, and SEN. Among these features, Toe-R SII showed lower values in PD than in HC, Toe-R NEG also decreased in PD, whereas Toe-R EN increased in PD. These opposite directions indicate that PD-related toe-region alterations were expressed as coordinated changes in information intensity, entropy, and non-Gaussian signal structure rather than as a uniform increase or decrease across all descriptors. The representative toe-region signal profile further showed that the HC and PD curves shared a similar loading pattern over the normalized gait cycle, but the PD group exhibited broader dispersion around the loading and unloading phases.

The compact visualization also identified Forefoot-L SII as one of the strongest discriminative features. The walking-recording level distribution showed lower SII values in PD than in HC, and the corresponding forefoot signal profile demonstrated a comparable overall waveform with visible group-level differences in the mid-to-late stance phase. These findings suggest that the most discriminative information-theoretic alterations were concentrated in the toe and forefoot regions, while the heatmap provides a broader overview of weaker but spatially distributed changes across the plantar surface.

**Figure 6:**
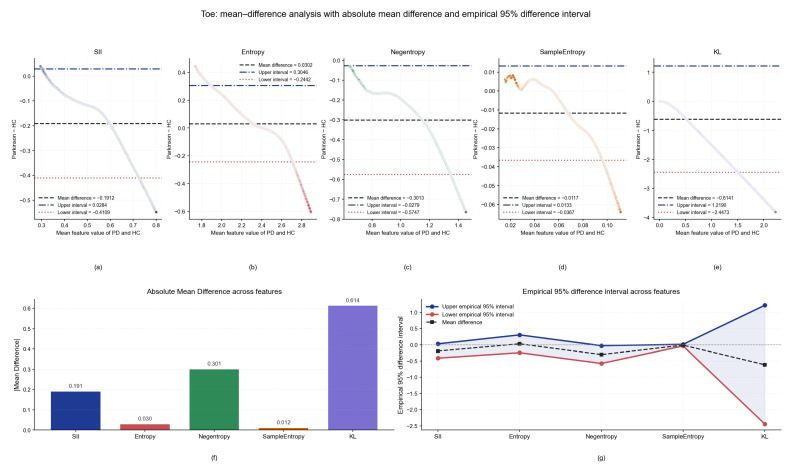
Mean–difference analysis of toe-region information-theoretic features. (**a**–**e**) Mean–difference plots for SII, EN, NEG, SEN, and KL, respectively. (**f**) Absolute mean difference across features. (**g**) Mean HC–PD difference with empirical 95% difference intervals.

To further quantify the magnitude and dispersion of toe-region differences, mean–difference analysis was performed, as shown in Figure 6. Figure 6a–e show the feature-specific HC–PD differences for SII, EN, NEG, SEN, and KL against the corresponding mean feature values of the two groups. The semi-transparent background points and their smoothed trajectory represent the walking-recording level mean–difference distribution for each feature, where the horizontal axis denotes the mean feature value of the HC and PD groups, and the vertical axis denotes the PD–HC difference. The black dashed line represents the mean PD–HC difference, while the blue dash-dotted and red dotted lines represent the upper and lower limits of the empirical 95% difference interval, respectively. The empirical 95% difference intervals were calculated from walking-recording level PD–HC differences after cycle-level features were aggregated within each recording file; therefore, they reflect between-recording variability rather than within-recording gait-cycle variability.

The absolute mean difference was largest for KL (0.614), followed by NEG (0.301) and SII (0.191), whereas EN (0.030) and SEN (0.012) showed smaller differences, as summarized in Figure 6f. Figure 6g further shows that the empirical 95% difference intervals were wider for KL and NEG, indicating larger between-recording variability in these features. These results suggest that KL, NEG, and SII are the most discriminative toe-region features for separating HC and PD groups.

### 3.2. Robustness and Computational Cost Analysis of Information-Theoretic Features

To evaluate the reliability of the region-specific information-theoretic plantar features, covariate-adjusted group comparison, clinical-severity association analysis, parameter-stability analysis, and computational cost analysis were performed.

**Figure 7:**
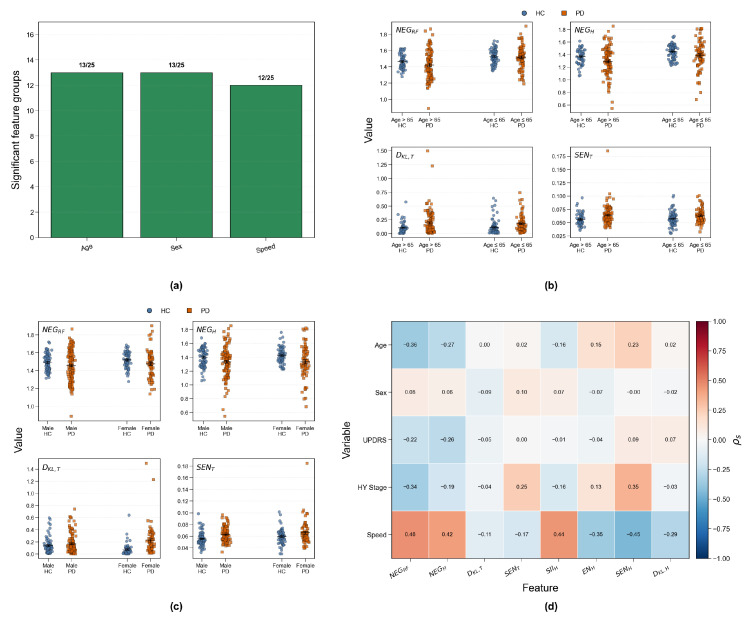
Covariate-adjusted group comparison and clinical-severity association analysis of representative region–descriptor information-theoretic plantar feature groups. (**a**) Number of significant region–descriptor feature groups retained after adjustment for age, sex, and walking speed. Each group was defined by one plantar region and one information-theoretic descriptor category. (**b**) Age-stratified distributions of representative features. (**c**) Sex-stratified distributions of representative features. (**d**) Spearman correlations between representative features and demographic, walking-speed, and clinical-severity variables. Correlations involving UPDRS and Hoehn–Yahr stage were calculated within the PD group.

As shown in Figure 7a, the analysis was summarized using 25 region–descriptor feature groups, defined by five plantar regions and five information-theoretic descriptor categories: heel, rearfoot, midfoot, forefoot, and toe; and SII, EN, NEG, SEN, and KL. Thus, the denominator of 25 represents the number of region–descriptor groups rather than the total number of original feature columns.

For the HC–PD group comparison, covariate-adjusted analysis was performed using age, sex, and walking speed, because these variables were available and clearly defined in both healthy controls and patients with Parkinson’s disease. After adjustment for age, sex, and walking speed, 13/25, 13/25, and 12/25 feature groups remained significant, respectively.

Clinical-severity association analysis was conducted within the PD group using UPDRS and Hoehn–Yahr stage. These variables were treated as PD clinical-severity indicators. Spearman correlation analysis was used to evaluate the associations between representative information-theoretic plantar features and clinical severity.

Figure 7b,c show that representative features retained HC–PD distributional differences across age and sex strata. Figure 7d shows weak to moderate correlations between representative features and demographic, walking-speed, and clinical-severity variables. The correlations involving UPDRS and Hoehn–Yahr stage represent PD-group clinical-severity associations.

Table 1 summarizes the parameter-stability analysis. The number of significant features was unchanged across normalization settings. Five-region and sensor-level representations retained similar proportions of significant features. Both 10 and 12 histogram bins retained 10/33 significant distributional features. Sample entropy computation remained below 0.001 s across the tested tolerance settings.

Table 2 reports the computational cost averaged at the subject level. The first 10 recordings corresponded to 6 subjects. Walking-recording level values were first averaged within each subject, followed by subject-level averaging. The mean signal duration was 121.17 s, the mean information-theoretic feature extraction time was 60.43 s, the mean ITFS-RM three-dimensional feature-space construction time was 0.346 ms, and the mean full computation time was 61.05 s.

Overall, Figure 7 and Table 1 and Table 2 show that the main HC–PD information-theoretic feature differences were not fully explained by age, sex, or walking speed. The PD-group clinical-severity association analysis further indicated that some representative plantar information-theoretic features showed weak to moderate associations with UPDRS or Hoehn–Yahr stage. The default settings, including sensor-wise maximum normalization, five-region plantar partitioning, 10-bin histogram estimation, and r=0.2×SD for sample entropy, showed stable results. The computational cost analysis further showed that the adopted feature-extraction procedure and final ITFS-RM three-dimensional feature-space construction were computationally feasible.

### 3.3. ITFS-RM Performance and Three-Dimensional Rule Structure

ITFS-RM was modeled and evaluated under a subject-grouped setting. The complete dataset included 306 walking-recording files from 165 subjects. The training set contained 226 recordings from 115 subjects, and the independent test set contained 80 recordings from 50 subjects. All walking-recording files from the same subject were kept within the same data subset. The model configuration, retained three-dimensional feature spaces, cascade rules, and decision thresholds were determined using the training set and were then fixed before being applied to the independent test set.

Table 3 summarizes the walking-recording level and subject-aggregated performance of ITFS-RM under subject-grouped evaluation. On the independent test set, the walking-recording level evaluation achieved an accuracy of 0.8250, precision of 0.8448, recall of 0.9074, F1-score of 0.8750, macro F1-score of 0.7917, and AUC of 0.7853. After subject-level aggregation, ITFS-RM achieved an accuracy of 0.7400, precision of 0.7273, recall of 0.8571, F1-score of 0.7869, macro F1-score of 0.7268, and AUC of 0.7638. The subject-grouped five-fold cross-validation and LOSO results were within a comparable performance range, indicating that the rule model retained stable discriminative ability under subject-grouped evaluation.

**Figure 8:**
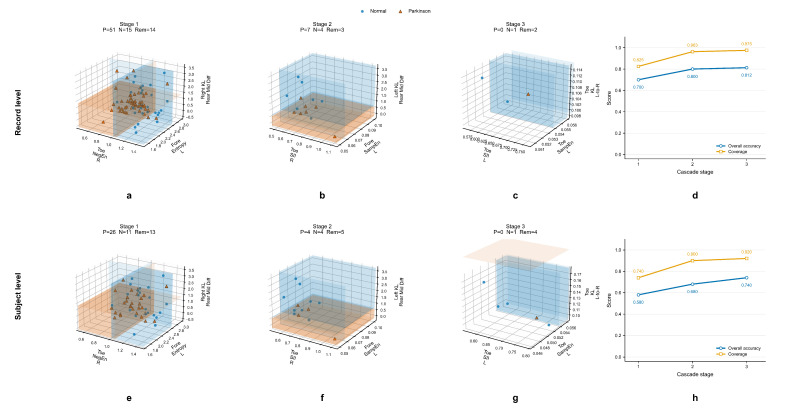
Walking-recording level and subject-aggregated ITFS-RM cascade rule structure. (**a**–**c**) Three retained ITFS-RM feature spaces at the recording level, corresponding to $S_{1}=\left(NEG_{T,r},EN_{FF,l},\Delta KL_{r}^{RF,MF}\right)$, $S_{2}=\left(SII_{T,r},SEN_{FF,l},\Delta KL_{l}^{RF,MF}\right)$, and $S_{3}=\left(SII_{T,l},SEN_{T,l},KL_{T}^{l\to r}\right)$, respectively. (**d**) Cumulative accuracy and coverage across walking-recording level cascade stages. (**e**–**g**) Corresponding retained feature spaces at the subject-aggregated level. (**h**) Cumulative accuracy and coverage across subject-aggregated cascade stages. P, N, and Rem denote the numbers of samples assigned as Parkinson’s disease, assigned as normal, and remaining unresolved, respectively. The transparent blue and orange regions indicate the class-specific clean regions for the Normal and Parkinson classes, respectively.

Figure 8 shows the three retained three-dimensional information-theoretic feature spaces and the cascade rule trajectories in the independent test cohort. Using the notation defined in Section 2, the retained ITFS-RM feature spaces were written as

(50) $$S_{1}=\left(NEG_{T,r},\;EN_{FF,l},\;\Delta KL_{r}^{RF,MF}\right),$$

(51) $$S_{2}=\left(SII_{T,r},\;SEN_{FF,l},\;\Delta KL_{l}^{RF,MF}\right),$$

(52) $$S_{3}=\left(SII_{T,l},\;SEN_{T,l},\;KL_{T}^{l\to r}\right),$$

where $S_{1}$, $S_{2}$, and $S_{3}$ denote the first, second, and third retained three-dimensional feature spaces, respectively. Here, *T*, FF, RF, and MF denote toe, forefoot, rearfoot, and midfoot, respectively, and *l* and *r* denote the left and right foot. The term $\Delta KL_{F}^{RF,MF}$ denotes the signed directional KL difference between rearfoot and midfoot on foot side *F*, and $KL_{T}^{l\to r}$ denotes the left-to-right KL divergence of the toe region.

At the recording level, $S_{1}$ assigned 51 PD recordings and 15 HC recordings, leaving 14 recordings unresolved. The cumulative coverage and overall accuracy were 0.825 and 0.700, respectively. The second feature space $S_{2}$ further assigned 7 PD recordings and 4 HC recordings, leaving 3 recordings unresolved. The cumulative coverage increased to 0.963, and the overall accuracy increased to 0.800. The third feature space $S_{3}$ further assigned 1 HC recording, resulting in a final coverage of 0.975 and an overall accuracy of 0.812.

At the subject-aggregated level, the same retained cascade structure was evaluated after aggregating walking-recording level rule assignments within each subject. The first feature space $S_{1}$ assigned 26 PD subjects and 11 HC subjects, leaving 13 subjects unresolved. The cumulative coverage and overall accuracy were 0.740 and 0.580, respectively. The second feature space $S_{2}$ further assigned 4 PD subjects and 4 HC subjects, increasing the cumulative coverage and accuracy to 0.900 and 0.680. The third feature space $S_{3}$ assigned 1 additional HC subject, yielding a final coverage of 0.920 and an overall accuracy of 0.740.

Table 4 reports the stage-wise walking-recording level and subject-aggregated assignments in the independent test cohort. The first feature space $S_{1}$ captured the dominant separation structure. The second feature space $S_{2}$ further resolved samples associated with forefoot temporal complexity and rearfoot–midfoot distributional transition differences. The third feature space $S_{3}$ refined the remaining assignments using toe-region SII, toe-region sample entropy, and toe left-to-right KL divergence. Toe-related descriptors appeared across all cascade stages, and KL-based directional features were retained in each stage, indicating that distal-foot information organization, inter-foot asymmetry, and adjacent-region loading-transition differences jointly contributed to the ITFS-RM decision structure.

Table 5 summarizes the explicit decision rules retained by the final ITFS-RM model. The walking-recording level coverage values were calculated during the development walking-recording level rule-search process, whereas the subject-aggregated coverage values were obtained by applying the retained rules to the independent subject-aggregated cohort. In the first stage, the HC rule was defined by higher $NEG_{T,r}$ and lower $EN_{FF,l}$, whereas the PD rule was defined by lower $NEG_{T,r}$ and lower $\Delta KL_{r}^{RF,MF}$. In the second stage, the HC rule was determined by the joint threshold of $SEN_{FF,l}$ and $\Delta KL_{l}^{RF,MF}$, whereas the PD rule was defined by an interval of $\Delta KL_{l}^{RF,MF}$. In the third stage, $SEN_{T,l}$ and $KL_{T}^{l\to r}$ formed the final HC and PD rules, respectively.

These results show that the discriminative process of ITFS-RM was not determined by a single information-theoretic descriptor but by rule regions formed across multiple local three-dimensional feature spaces. Toe-region features, forefoot temporal-complexity descriptors, and KL-based plantar asymmetry features constituted the main interpretable structure of the model. Unlike conventional classifiers that only provide class labels or probability scores, ITFS-RM jointly reports feature combinations, numerical boundaries, and cascade assignment trajectories, thereby linking HC–PD separation to region-specific information-theoretic alterations in plantar loading.

### 3.4. Comparative Classification, Cross-Validation, and Ablation Analysis at the Gait-Cycle and Walking-Recording Levels

To assess the discriminative capability of the proposed information-theoretic feature representation, eight conventional machine learning classifiers were evaluated at both gait-cycle and walking-recording levels, including LogisticRegression, DecisionTree, AdaBoost, GradientBoosting, RandomForest, SVM, MLP, and KNN. Accuracy, precision, recall, F1-score, and AUC were used as evaluation metrics. The gait-cycle level results are summarized in Table 6.

At the gait-cycle level, KNN achieved the best overall test performance, with an accuracy of 0.9668, an F1-score of 0.9763, and an AUC of 0.9876. RandomForest obtained the highest recall and AUC on the test set, indicating strong sensitivity to PD gait cycles, whereas SVM achieved the highest precision, reflecting a lower false-positive tendency. MLP also showed competitive performance, with a test accuracy of 0.9617 and an F1-score of 0.9725. In contrast, LogisticRegression and AdaBoost showed lower overall performance, suggesting that the gait-cycle level feature space contains nonlinear discriminative structures that are not fully captured by simpler linear or weak-learner-based models.

The walking-recording level classification results are reported in Table 7.

At the walking-recording level, MLP achieved the best overall test performance, with an accuracy of 0.9344, precision of 0.9524, recall of 0.9524, and F1-score of 0.9524. GradientBoosting produced the highest test recall, indicating strong sensitivity for PD walking-recording level identification. RandomForest achieved the highest walking-recording level test AUC, suggesting better ranking ability across decision thresholds. These results show that although KNN was most effective for gait-cycle level discrimination, MLP provided a more balanced decision boundary when the classification unit was a complete walking-recording file.

#### 3.4.1. Five-Fold Cross-Validation and LOSO Validation

To further evaluate the stability of the proposed region-specific information-theoretic feature representation under repeated data partitioning, five-fold cross-validation was performed at both gait cycle and walking-recording levels. In contrast to the fixed validation/test evaluation reported above, this analysis focuses on the mean performance and 95% confidence interval across different folds. The gait-cycle level evaluation was used to assess the stability of local sample-wise discrimination, whereas the walking-recording level evaluation was used to assess the stability of complete-recording discrimination. Table 8 summarizes the cross-validation results at both evaluation levels.

At the gait-cycle level, KNN achieved the highest mean accuracy and F1-score, with values of 0.9658 and 0.9756, respectively. This finding is consistent with the fixed test-set evaluation, where KNN also showed the best overall gait-cycle level performance. RandomForest obtained the highest mean recall and AUC, reaching 0.9892 and 0.9887, respectively, further indicating its strong sensitivity to PD gait-cycle samples. SVM and MLP achieved high precision, suggesting their advantage in reducing false-positive predictions.

At the walking-recording level, the overall performance was lower than that at the gait-cycle level. SVM achieved the highest mean accuracy, precision, and F1-score, with values of 0.8497, 0.8903, and 0.8932, respectively. RandomForest obtained the highest mean recall and AUC, reaching 0.9344 and 0.9150, respectively, indicating strong sensitivity to PD recordings and favorable threshold-independent discrimination. The mean performance of MLP was close to that of SVM and RandomForest, which is consistent with its strong walking-recording level performance under the fixed test-set evaluation.

**Figure 9:**
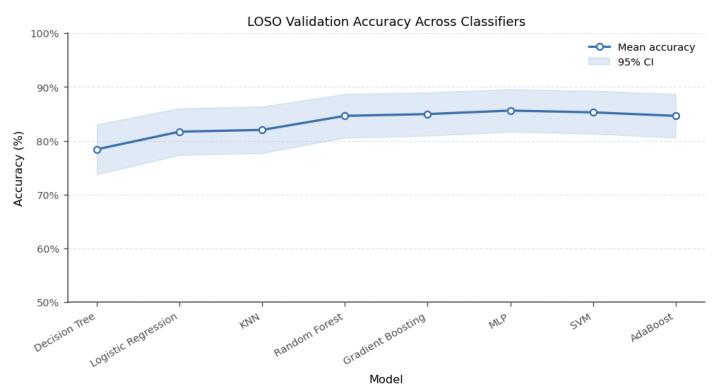
Leave-one-subject-out validation accuracy of different classifiers. Values are reported as mean accuracy with 95% confidence interval.

In addition to five-fold cross-validation, leave-one-subject-out (LOSO) validation was performed to further assess subject-independent generalization. In LOSO validation, one subject was left out for testing in each iteration, and the remaining subjects were used for model training. Since each test fold corresponded to an unseen subject, LOSO provides a stricter evaluation of individual-level generalization. Figure 9 reports the LOSO accuracy of each classifier.

The LOSO results showed that MLP achieved the highest mean accuracy of 0.8562, followed by SVM with 0.8529 and GradientBoosting with 0.8497. RandomForest and AdaBoost obtained the same mean accuracy of 0.8464. Compared with the walking-recording level five-fold cross-validation results, LOSO produced more conservative accuracy values, which is expected because each test iteration involved a completely unseen subject. Nevertheless, the LOSO results remained consistent with the cross-validation trend, indicating that nonlinear classifiers, especially MLP, SVM, GradientBoosting, and RandomForest, maintained relatively stable subject-independent performance.

#### 3.4.2. Regional and Information-Theoretic Feature-Type Ablation Analysis

To further evaluate the relative contribution of different plantar regions and information-theoretic feature types, ablation analysis was performed at both gait-cycle and walking-recording levels. For each classifier, one plantar region or one information-theoretic feature type was removed, and the resulting performance decrease was calculated relative to the complete feature set of the same classifier. To avoid overinterpreting small performance fluctuations from individual classifiers, the ablation results were summarized using a rank-based ablation importance score (AIS).

For each classifier, the ablated components were ranked according to the performance decrease after removal, where rank 1 indicated the largest performance decrease and therefore the strongest contribution. The ranks were then averaged across classifiers and converted into AIS as follows:

(53) $$AIS=\frac{N-\bar{R}}{N-1}\times 100,$$

where AIS denotes the ablation importance score, $\bar{R}$ is the mean rank across classifiers, and *N* is the number of ablated components within the same ablation group. In this study, N=5 for both regional ablation and information-theoretic feature-type ablation. A higher AIS indicates a stronger relative contribution to classification performance. For tied ranks, the average rank was used.

As shown in Table 9, the toe region obtained the highest AIS at both gait-cycle and walking-recording levels. At the gait-cycle level, toe-region removal ranked as the most influential regional ablation for both accuracy and F1-score, with AIS values of 100.00. This indicates that toe-region features provided the strongest regional contribution to local gait-cycle discrimination. Midfoot and heel features showed moderate gait-cycle level contributions, whereas rearfoot and forefoot produced lower AIS values.

At the walking-recording level, the toe region also achieved the highest AIS for both accuracy and F1-score, with values of 65.62. Rearfoot showed the second-highest AIS at the walking-recording level, reaching 57.81 for both metrics. These results suggest that toe-region features were consistently important across evaluation levels, while rearfoot information became more relevant when classification was performed using complete walking-recording representations.

As shown in Table 10, the information-theoretic feature-type contribution pattern differed between gait-cycle level and walking-recording level classification. At the gait-cycle level, Entropy achieved the highest AIS for both accuracy and F1-score, with values of 95.31 and 93.75, respectively. Negentropy ranked second, with AIS values of 79.69 for accuracy and 81.25 for F1-score. These results indicate that local gait-cycle discrimination relied strongly on pressure-state uncertainty and non-Gaussian distributional organization.

At the walking-recording level, the contribution pattern became more balanced across feature types. For accuracy-based AIS, Sample Entropy achieved the highest score of 57.81, followed by Negentropy and SII. For F1-based AIS, Negentropy achieved the highest score of 57.81, followed by SII and Sample Entropy. These results suggest that walking-recording level classification benefited more from temporal irregularity, non-Gaussian distributional structure, and self-information fluctuation. Entropy and KL showed lower walking-recording level AIS values, indicating that their independent ranking contribution was relatively smaller in the averaged analysis, although they may still provide complementary information when combined with other feature types.

Taken together, the ablation results show that the relative contribution of plantar regions and information-theoretic feature types was evaluation-level dependent. The toe region was consistently important at both gait cycle and walking-recording levels, supporting its role in capturing terminal stance and push-off abnormalities in PD gait. Rearfoot features became more influential at the walking-recording level, suggesting that load transfer and stance-phase control contributed to complete-walking-recording level classification. At the feature-type level, Entropy and Negentropy were more influential for gait-cycle level classification, whereas Negentropy, Sample Entropy, and SII contributed more strongly to walking-recording level classification. These findings support the use of both regional plantar organization and multiple information-theoretic feature types rather than relying on a single anatomical region or a single descriptor group.

### 3.5. Subject-Independent and Subject-Balanced Generalization Evaluation

To evaluate the generalization ability of the region-specific information-theoretic features to unseen subjects, a series of subject-independent and subject-balanced experiments was conducted. These experiments included subject-independent walking-recording level classification, subject-grouped cross-validation, leave-one-subject-out validation, cross-subject ablation analysis, subject-balanced gait-cycle aggregation, and subject-cycle feature ablation. The independent test set was used only for final performance reporting and was not involved in training. This prevented repeated walking recordings from the same subject from appearing simultaneously in model development and performance evaluation, thereby providing a stricter assessment of the model’s generalization ability to new subjects.

#### 3.5.1. Subject-Independent Test-Set Performance

Complete walking recordings were used as the analysis unit to compare the performance of different classifiers on the subject-independent test set. Accuracy, precision, recall, F1-score, and AUC were used as evaluation metrics. As shown in Table 11, GradientBoosting and MLP both achieved the highest accuracy of 0.8475, indicating that the region-specific information-theoretic features had favorable cross-subject discriminative ability. MLP obtained the highest AUC of 0.9119 and the highest F1-score of 0.8966, suggesting strong overall discriminative ability in distinguishing healthy control and Parkinson’s disease walking recordings. GradientBoosting achieved the highest precision of 0.9000, indicating high reliability among recordings predicted as Parkinson’s disease. RandomForest, SVM, and AdaBoost also achieved accuracies above 0.81, showing that the discriminative value of the region-specific information-theoretic features was preserved across multiple model structures.

Compared with ordinary walking-recording level random splitting, the subject-independent test setting more closely reflects the practical scenario in which training and test subjects do not overlap.

#### 3.5.2. Subject-Grouped Five-Fold Cross-Validation

To further evaluate model stability during development, subject-grouped five-fold cross-validation was performed within the development set. In each fold, the subject identifier was used as the grouping variable, ensuring that all recordings from the same subject were kept either in the training fold or in the validation fold. Table 12 reports the average five-fold cross-validation performance.

Table 12 shows that the classifiers achieved moderate to high performance under subject-grouped cross-validation. KNN obtained the highest accuracy of 0.7664 and the highest F1-score of 0.8288, while AdaBoost achieved a comparable accuracy of 0.7660. SVM achieved the highest AUC of 0.8381 and the highest precision of 0.8785. These results indicate that the region-specific information-theoretic features provided stable classification signals when model selection was performed using only development subjects. Subject-grouped five-fold cross-validation was used to estimate development-stage robustness rather than to replace the independent test-set evaluation. Together with Table 11, these results show that the proposed features retained discriminative ability in both internal grouped validation and independent held-out testing.

#### 3.5.3. Leave-One-Subject-Out Validation

As a stricter subject-level generalization assessment, leave-one-subject-out (LOSO) validation was further performed. In each round, all recordings from one subject were held out as the test samples, whereas the remaining subjects were used for model training. Predictions from all rounds were pooled to calculate the overall performance. Table 13 summarizes the pooled prediction results under the LOSO setting.

Under LOSO validation, RandomForest achieved the best overall performance, with an accuracy of 0.8137, precision of 0.8985, F1-score of 0.8613, and AUC of 0.8700. KNN and RandomForest both obtained a recall of 0.8271, indicating favorable recognition of PD recordings. By contrast, DecisionTree showed substantially lower performance, suggesting that a single decision tree was less stable under high inter-subject heterogeneity. Because LOSO evaluates each subject as an unseen individual in turn, it is more sensitive to cross-subject variability. The strong AUC of RandomForest under this setting further supports the robustness of the region-specific information-theoretic features in subject-independent evaluation.

#### 3.5.4. Cross-Subject Ablation Analysis

To identify the contribution of different information-theoretic feature types and anatomical plantar regions to cross-subject classification, ablation analysis was performed using GradientBoosting as the reference model. Since the information-type and region-level ablation analyses used the same full-feature model as the baseline, the two ablation settings were summarized in a single table. The change in accuracy after removing each feature group was also reported to facilitate comparison of feature-group importance.

As shown in Table 14, at the information-type level, removing SII or KL reduced the accuracy from 0.8475 to 0.7627, corresponding to a decrease of 0.0848. Removing Negentropy reduced the accuracy to 0.7797, corresponding to a decrease of 0.0678. Removing Entropy or Sample Entropy also caused performance decreases of different magnitudes. These results indicate that multiple information-theoretic descriptors provided complementary information, with SII, KL, and Negentropy having stronger effects on subject-independent test performance.

At the plantar-region level, removing midfoot caused the largest performance decrease, reducing the accuracy from 0.8475 to 0.7966. Removing heel, forefoot, or toe resulted in an accuracy of 0.8136, whereas removing rearfoot resulted in an accuracy of 0.8305 and had a relatively smaller effect. Overall, cross-subject walking-recording level classification was not determined by a single plantar region but relied on information-theoretic representations from multiple anatomical regions.

#### 3.5.5. Subject-Balanced Cycle Holdout Repeated Evaluation

In addition to complete-walking-recording level classification, this study further constructed a subject-balanced multi-cycle evidence aggregation strategy at the gait-cycle level to reduce potential sample-weight bias caused by unequal numbers of available gait cycles across subjects. In each repeated experiment, the same number of gait cycles was sampled from the gait-cycle pool of each subject. Cycle level predictions were first obtained and then aggregated at the subject level to generate the final decision.

Table 15 reports the results of the subject-balanced 50-cycle holdout repeated experiments. The mean and 95% confidence interval reflect performance stability under repeated sampling, whereas the best single-run accuracy describes the highest accuracy achieved by each classifier across repeated experiments.

As shown in Table 15, GradientBoosting achieved the highest mean accuracy of 0.8655 with a 95% CI of [0.8606, 0.8703] and the highest F1-score of 0.8723. RandomForest achieved a mean accuracy of 0.8618 and obtained the highest AUC of 0.9015, as well as one of the highest best single-run accuracies of 0.9091. AdaBoost also achieved an AUC of 0.8992, approaching the performance of RandomForest. These results indicate that subject-balanced multi-cycle evidence aggregation produced more stable classification performance than isolated cycle- or walking-recording level decisions.

Although DecisionTree, MLP, and SVM showed high recall, their accuracy or precision was relatively limited, suggesting a tendency toward PD-class predictions. By contrast, GradientBoosting and RandomForest achieved a better balance among accuracy, F1-score, and AUC and were therefore more suitable as representative models in the subject-balanced cycle-aggregation framework.

#### 3.5.6. Grouped Cross-Validation and LOSO Robustness of the Subject-Cycle Model

Because subject-cycle grouped five-fold cross-validation and LOSO validation used the same representative model, the two robustness-validation results are reported together. GradientBoosting was used as the representative classifier in both settings to evaluate the stability of the multi-cycle aggregation strategy under different subject-level validation protocols.

Table 16 shows that GradientBoosting achieved an accuracy of 0.7695, F1-score of 0.8083, and AUC of 0.8267 in subject-grouped five-fold cross-validation. Under LOSO cross-validation, the accuracy was 0.7212, the F1-score was 0.7553, and the AUC was 0.8475. Although LOSO produced a lower accuracy than five-fold cross-validation, its AUC remained relatively high, indicating stable ranking ability across unseen subjects.

The subject-cycle LOSO setting is stricter than ordinary holdout testing because each round requires prediction on a previously unseen individual. These results indicate that multi-cycle evidence aggregation improves the usability of cycle level predictions, while cross-subject heterogeneity remains an important factor affecting final accuracy.

#### 3.5.7. Subject-Cycle Feature Ablation Analysis

To further interpret the discriminative sources of the subject-cycle aggregation model, feature ablation analysis was performed on the GradientBoosting model by removing either one information-theoretic feature type or one anatomical plantar region. For direct comparison, both ablation settings were summarized together, and the accuracy change relative to the baseline model was reported.

Table 17 shows that Negentropy was one of the most critical information-theoretic feature types in the subject-cycle aggregation framework. Removing Negentropy reduced the accuracy from 0.8655 to 0.6717, corresponding to a decrease of 0.1938. Removing KL reduced the accuracy to 0.7576, corresponding to a decrease of 0.1079. Removing Entropy and Sample Entropy caused decreases of 0.0423 and 0.0453, respectively, whereas removing SII had a smaller effect. These results indicate that multiple information-theoretic features describing non-Gaussianity, distributional discrepancy, and signal complexity jointly supported the discriminative performance of cycle level evidence aggregation.

At the plantar-region level, the toe region had the strongest influence on the model. Removing toe reduced the accuracy to 0.6667, corresponding to a decrease of 0.1988. Forefoot had the second-largest effect, with an accuracy of 0.7798 after removal. Removing midfoot, rearfoot, and heel also caused performance decreases of different magnitudes, although the effects were smaller.

### 3.6. Comparison with Deep Learning Based on Information-Theoretic Features and Plantar Insole Sensor Signals

To further assess the effectiveness of the proposed information-theoretic feature representation, the best-performing conventional classifiers were compared with feature-level deep learning baselines and plantar insole sensor signal baselines. For the gait-cycle level comparison, the best conventional result was obtained by KNN using the proposed information-theoretic features. For the walking-recording level comparison, the best conventional result was obtained by MLP using the proposed information-theoretic features. For the subject-independent subject-aggregated comparison, the best conventional result was obtained by GradientBoosting using the proposed information-theoretic features.

The deep learning and plantar signal baseline models were implemented using the settings described in Section 2.

As shown in Table 18, the proposed information-theoretic feature representation achieved the highest test accuracy in the gait-cycle level, walking-recording level, and subject-independent subject-aggregated comparisons. At the gait-cycle level, KNN with the proposed features achieved an accuracy of 0.9668 and an F1-score of 0.9763, outperforming all feature-level deep learning baselines. Among these deep learning models, CNN–BiLSTM achieved the highest gait-cycle level accuracy of 0.8303.

At the walking-recording level, MLP with the proposed information-theoretic features achieved the highest accuracy of 0.9344 and F1-score of 0.9524. Among the feature-level deep learning baselines, CNN achieved the best walking-recording level accuracy of 0.8689 and F1-score of 0.9048. For plantar insole sensor signal input, CNN achieved the highest accuracy of 0.8197 and the highest AUC of 0.9511. Although the plantar insole sensor signal CNN showed strong ranking ability, its accuracy and F1-score remained lower than those obtained using the proposed information-theoretic features.

At the subject-independent subject-aggregated level, GradientBoosting with the proposed information-theoretic features achieved the highest accuracy of 0.8655 and F1-score of 0.8723. Among the feature-level deep learning models based on subject-aggregated information-theoretic features, MLP achieved the best performance, with an accuracy of 0.7576, precision of 0.7083, recall of 0.9444, F1-score of 0.8095, and AUC of 0.6815. These results indicate that feature-level deep learning did not exceed the best conventional-classifier performance obtained with the proposed feature representation under subject-independent subject-aggregated evaluation.

These results indicate that directly learning from plantar insole sensor signals or applying deep learning to the extracted feature representation did not consistently outperform the proposed information-theoretic feature representation combined with conventional classifiers. The proposed feature representation provided a compact and discriminative input space, leading to stronger test-set classification performance at gait-cycle, walking-recording, and subject-independent subject-aggregated levels. The subject-independent comparison further suggests that compact information-theoretic representations remain suitable for small-sample unseen-subject evaluation.

### 3.7. Accuracy Comparison with Studies Based on Plantar Insole Sensor Signals for PD Classification

As shown in Table 19, representative insole sensor-based PD classification studies using the same public gait dataset have adopted different feature domains and classifier models, including neural network (NN), Support Vector Machine (SVM), linear discriminant analysis (LDA), hybrid ConvNet–Transformer (HCT), convolutional neural network (CNN), long short-term memory with particle swarm optimization and genetic optimization (LSTM-PSOGO), back-propagation artificial neural network (BPANN), Multi-Layer Perceptron (MLP), recurrent neural network (RNN), and K-Nearest Neighbors (KNN).

For the proposed method, three results are reported according to the corresponding evaluation setting. Under the subject-independent subject-aggregated evaluation, the proposed region-specific information-theoretic features achieved an accuracy of 86.55% using GradientBoosting. At the walking-recording level, the proposed features achieved 93.44% accuracy using MLP. At the gait-cycle level, the proposed features achieved 96.68% accuracy using KNN.

Within the context of the same public PD gait dataset, the proposed method achieved accuracy within the upper range of reported results. The subject-independent subject-aggregated result provides a more conservative evaluation of unseen-subject generalization, whereas the walking-recording level and gait-cycle level results characterize discriminative performance under their corresponding sample definitions. These findings suggest that region-specific information-theoretic features provide a competitive and interpretable representation of plantar insole sensor signals for HC–PD classification.

## 4. Discussion

### 4.1. Main Findings Across Gait-Cycle, Recording, and Subject-Wise Analyses

This study proposed a region-specific information-theoretic framework for Parkinsonian gait assessment using wearable plantar insole signals. The proposed framework transforms plantar-pressure time series into regional features that characterize probabilistic uncertainty, self-information fluctuation, non-Gaussian organization, temporal irregularity, and directional distributional discrepancy. By integrating plantar regional reorganization, gait-cycle segmentation, information-theoretic feature extraction, KL-based distributional modeling, supervised classification, ablation analysis, and the ITFS-RM, the framework provides both predictive and interpretable evidence for HC–PD separation.

The experimental results support three main findings. First, PD-related plantar-pressure differences were spatially heterogeneous rather than uniformly distributed across the plantar surface. Second, different information-theoretic features captured complementary aspects of plantar-signal organization, suggesting that PD-related gait alterations cannot be sufficiently characterized by pressure amplitude or low-order statistical features alone. Third, the proposed features remained discriminative across gait-cycle level, walking-recording level, subject-independent, subject-grouped cross-validation, LOSO, ablation, and baseline-comparison experiments, indicating that the extracted features captured reproducible discriminative patterns under the evaluated settings.

These evaluation levels should be interpreted according to their sample definitions. Gait-cycle level analysis reflects local short-term separability within plantar-pressure dynamics. Walking-recording level analysis reflects complete acquisition-level separability, where each recording preserves a specific walking condition, speed, or task state. This level is meaningful because walking speed and task condition can alter plantar-pressure distribution, temporal regularity, and regional load transfer; therefore, each complete recording may contain condition-specific feature information. Subject-wise analysis further evaluates whether the learned feature patterns transfer to individuals whose recordings were not used during model development.

The performance pattern was consistent with this hierarchy. KNN achieved a gait-cycle level test accuracy of 0.9668, and MLP achieved a walking-recording level test accuracy of 0.9344, showing strong local and complete-recording separability. Under subject-independent evaluation, the estimates were lower but more conservative: GradientBoosting and MLP achieved an accuracy of 0.8475 on the held-out test set, while the subject-balanced 50-cycle holdout evaluation reached a mean accuracy of 0.8655 with GradientBoosting. Under LOSO validation, RandomForest achieved an accuracy of 0.8137 at the pooled prediction level, and the subject-cycle LOSO model obtained an accuracy of 0.7212. This decrease is expected because subject-wise evaluation reduces the influence of subject-specific similarity and increases the effect of inter-subject gait heterogeneity.

### 4.2. Regional Plantar Information and Biomechanical Interpretation

The region-specific analysis showed that discriminative information was mainly concentrated in the heel, midfoot, forefoot, and toe regions, with the toe and forefoot regions showing the most evident group-related differences. This spatial pattern is biomechanically plausible. The heel and rearfoot are closely related to initial contact and early loading, whereas the midfoot contributes to stance-phase support and load transfer. The toe region is involved in terminal stance and push-off, during which forward progression is generated through distal plantar loading and forefoot lever function [33]. Therefore, the strong contribution of toe-region features may indicate altered distal load transfer and push-off control in PD gait, rather than only global gait slowing or stride variability.

The compact walking-recording level visualization in Figure 5 supports this regional interpretation. The overview heatmap shows that HC–PD differences were distributed across multiple plantar region–foot-side combinations rather than being restricted to a single sensor or region. Among the most discriminative features, Toe-R SII, Forefoot-L SII, Toe-R NEG, and Toe-R EN showed clear group shifts. Toe-R SII and Toe-R NEG were lower in PD than in HC, whereas Toe-R EN was higher in PD. These opposite directions suggest coordinated changes in information fluctuation, entropy, and non-Gaussian organization in the toe region, rather than a uniform change across all descriptors.

The side-specific pattern in Figure 5 is compatible with the asymmetric nature of PD motor impairment. PD-related motor signs and bilateral coordination deficits may affect plantar information organization differently between the two feet. In the present data, stronger right-toe differences and a strong left-forefoot SII difference were observed. These findings should not be interpreted as a universal disease-side pattern because the dataset does not provide complete information on the clinically more affected side for every participant. Instead, they indicate that bilateral plantar information is not redundant and that side-specific regional features may capture complementary aspects of PD-related gait control.

The information-theoretic findings further suggest that PD-related plantar changes involve alterations in signal organization and distributional structure. KL divergence reflects directional distributional discrepancy between bilateral homologous regions or adjacent plantar regions and thus provides a representation of plantar-load redistribution and asymmetry. Negentropy characterizes deviation from Gaussian-like organization, indicating changes in the distributional structure of plantar loading. SII reflects the fluctuation of self-information and may describe the stability of plantar loading states. SEN captures temporal irregularity within regional pressure sequences. Together, these features suggest that PD-related plantar abnormalities involve not only changes in pressure magnitude but also changes in how plantar loading states are organized, redistributed, and repeated across gait cycles.

The toe-region mean–difference analysis in Figure 6 further shows that the largest absolute HC–PD mean difference was observed for KL (0.614), followed by NEG (0.301) and SII (0.191), whereas EN (0.030) and SEN (0.012) showed smaller mean differences. This result suggests that toe-region distributional discrepancy, non-Gaussian organization, and self-information fluctuation were the most prominent toe-related descriptors in the present data. The semi-transparent background points and smoothed curves in Figure 6a–e represent walking-recording level mean–difference distributions. The empirical 95% intervals reflect between-recording variability after cycle level features were aggregated within each recording file, rather than within-recording gait-cycle variability.

The use of negentropy does not assume that plantar-pressure signals are Gaussian. Plantar-pressure sequences are expected to show non-Gaussian characteristics because they include near-zero swing-phase values, stance-phase loading peaks, and asymmetric loading and unloading patterns. In this context, the Gaussian distribution is used as a maximum-entropy reference with matched variance, and negentropy quantifies the departure of the observed plantar distribution from this reference. Therefore, NEG should be interpreted as a contrast measure of distributional organization rather than as evidence that plantar loading follows a Gaussian model.

The ablation analyses were consistent with the regional and feature-level interpretation. At the gait-cycle and walking-recording levels, the toe region obtained the highest ablation importance score, indicating its stable contribution to local and complete-recording discrimination. In the subject-independent ablation analysis, removing SII or KL reduced the GradientBoosting accuracy from 0.8475 to 0.7627, while removing Negentropy reduced the accuracy to 0.7797. At the plantar-region level, removing midfoot caused the largest subject-independent decrease, reducing accuracy from 0.8475 to 0.7966. In the subject-balanced cycle aggregation analysis, removing toe reduced accuracy from 0.8655 to 0.6667, and removing Negentropy reduced accuracy to 0.6717. These results indicate that PD-related plantar information was distributed across both anatomical regions and information-theoretic descriptors, with toe-related and non-Gaussian features showing particularly stable contributions.

### 4.3. Subject-Wise Generalization and Data-Leakage Considerations

Data leakage is a central methodological concern in wearable gait classification when repeated recordings or repeated gait cycles from the same participant are treated as independent samples. In this situation, a model may partially learn subject-specific gait characteristics rather than disease-related patterns, and evaluation performance may be inflated if the test set contains recordings from individuals already represented during training. This concern is particularly relevant for public gait datasets in which some participants contribute multiple recordings under different walking conditions, speeds, or task states.

For this reason, gait-cycle level, walking-recording level, and subject-wise analyses should be considered complementary rather than interchangeable. The first two levels quantify feature separability under local-cycle and complete-recording sample definitions. They remain useful because different walking conditions and speeds can produce distinct plantar-pressure patterns and may reveal how information-theoretic descriptors behave across acquisition contexts. However, they should not be interpreted as direct estimates of clinical diagnostic performance when repeated recordings from the same subject exist. Subject-wise holdout, subject-grouped cross-validation, and LOSO validation provide more relevant estimates for unseen-subject assessment.

Among the subject-wise schemes, LOSO is the strictest individual-level validation because each subject is held out once and evaluated as an unseen individual. This setting most closely approximates clinical application, in which a trained model is applied to a new person whose recordings were unavailable during model development. The lower LOSO performance compared with gait-cycle level and walking-recording level performance is therefore expected and reflects the influence of inter-subject heterogeneity, unequal recording numbers, disease variability, and walking-condition differences. Accordingly, the LOSO accuracy of 0.8137 for RandomForest and the subject-cycle LOSO accuracy of 0.7212 should be interpreted as conservative evidence of generalization rather than as a contradiction of the higher sample-level results.

This distinction also informs the interpretation of ITFS-RM. High development-stage rule separability indicates that the selected three-dimensional feature spaces can define class-pure regions in the development data, but such values should not be treated as final generalization estimates. Greater emphasis should be placed on independent test, subject-grouped cross-validation, and LOSO results. Future optimization should therefore use nested subject-grouped model selection, limit rule complexity, apply regularization where appropriate, and validate retained rule spaces on independent cohorts.

### 4.4. Predictive Performance and Model-Level Evidence

The classification results demonstrate that the proposed information-theoretic representation is effective at both gait-cycle and walking-recording levels, but these two evaluation levels reflect different aspects of model behavior. Gait-cycle level evaluation contains more local samples and is therefore suitable for identifying repeated short-term discriminative patterns within plantar-pressure dynamics. The strong performance of KNN at this level suggests that gait cycles with similar class labels form locally coherent neighborhoods in the proposed information-theoretic feature space. The higher gait-cycle level accuracy should be interpreted as evidence of separability of gait abnormality at the local cycle level.

Walking-recording level evaluation is also necessary because each sample corresponds to one complete walking acquisition rather than to an isolated gait cycle. Different recordings may reflect different walking speeds, task states, or acquisition conditions, and these factors can affect plantar-pressure distribution, temporal regularity, and regional load transfer. Therefore, walking-recording level classification provides an additional view of complete-condition separability and helps evaluate whether the proposed features preserve discriminative information after cycle level information is aggregated within a recording. The strong walking-recording level performance of MLP suggests that complete-recording classification benefits from nonlinear integration of multiple regional and information-theoretic descriptors.

Subject-wise results provide a more conservative estimate of generalization. On the subject-independent held-out test set, GradientBoosting and MLP achieved the highest accuracy of 0.8475, and MLP achieved the highest AUC of 0.9119. In subject-grouped five-fold cross-validation, KNN obtained the highest accuracy of 0.7664 and the highest F1-score of 0.8288, while SVM achieved the highest AUC of 0.8381. Under LOSO validation, RandomForest achieved the best pooled performance, with an accuracy of 0.8137, F1-score of 0.8613, and AUC of 0.8700.

The subject-balanced 50-cycle holdout experiment further reduced sample-count bias caused by unequal numbers of available cycles. GradientBoosting achieved the highest mean accuracy of 0.8655 with a 95% confidence interval of 0.8606–0.8703, an F1-score of 0.8723, and an AUC of 0.8875. The grouped cross-validation result of the subject-cycle model was lower, with an accuracy of 0.7695 and AUC of 0.8267, and the LOSO result was further reduced to 0.7212 accuracy and 0.8475 AUC. This pattern indicates that stricter subject-wise validation produces more conservative estimates and should be prioritized when discussing unseen-subject assessment.

The four-decimal reporting format in the tables was retained to preserve numerical consistency across classifiers and experiments. Nevertheless, interpretation should not rely on differences at the fourth decimal place. Performance comparisons should focus on consistent trends across evaluation settings, confidence intervals where available, and whether the evaluation was performed at the gait-cycle, walking-recording, subject-independent, or LOSO level.

### 4.5. Interpretable Three-Dimensional Feature-Space Rules

A key contribution of this study is the ITFS-RM, which complements conventional classifier-based evaluation. Supervised classifiers demonstrate whether the proposed features are predictive, but they do not directly explain how HC and PD subjects are separated in the feature space. In contrast, ITFS-RM identifies class-pure regions in sequential three-dimensional information-theoretic feature spaces and provides explicit feature combinations, value ranges, and spatial rule boundaries. This design improves the transparency of the decision structure and facilitates interpretation of the regional information-theoretic feature space.

The retained ITFS-RM spaces provide a direct description of the HC–PD separation structure. The first retained space was $S_{1}=\left(NEG_{T,r},\;EN_{FF,l},\;\Delta KL_{r}^{RF,MF}\right)$, which combines right-toe non-Gaussian organization, left-forefoot entropy, and right rearfoot–midfoot directional KL transition. The second retained space was $S_{2}=\left(SII_{T,r},\;SEN_{FF,l},\;\Delta KL_{l}^{RF,MF}\right)$, which links right-toe self-information fluctuation, left-forefoot temporal irregularity, and left rearfoot–midfoot KL transition. The third retained space was $S_{3}=\left(SII_{T,l},\;SEN_{T,l},\;KL_{T}^{l\to r}\right)$, which focuses on left-toe information fluctuation, left-toe temporal irregularity, and bilateral toe asymmetry. These retained spaces indicate that the rule structure mainly relied on toe-region organization, forefoot signal complexity, and KL-based regional or bilateral asymmetry.

The explicit rules further clarify the separation mechanism. One HC-oriented rule in Stage 1 was defined by $NEG_{T,r}\geq 1.01484$ and $EN_{FF,l}\leq 2.62961$, whereas one PD-oriented rule in the same stage was defined by $NEG_{T,r}\leq 1.06573$ and $\Delta KL_{r}^{RF,MF}\leq 1.33196$. Additional rules involved $SEN_{FF,l}$, $\Delta KL_{l}^{RF,MF}$, $SEN_{T,l}$, and $KL_{T}^{l\to r}$. These boundaries show that HC–PD separation was not determined by a single threshold but by combinations of toe, forefoot, and KL-transition descriptors.

The subject-grouped ITFS-RM results should be interpreted with appropriate caution. The independent test accuracy was 0.8250 at the recording level and 0.7400 after subject aggregation. Under subject-grouped five-fold cross-validation, the corresponding accuracies were 0.7965 and 0.7565, while under LOSO, they were 0.7788 and 0.7304. These values are lower than development-stage rule-fitting values, but they provide a more realistic estimate of transferability to unseen subjects. Therefore, the main value of ITFS-RM lies in transparent rule discovery and interpretable feature-space separation, rather than in maximizing apparent training accuracy.

The interpretability of ITFS-RM is particularly relevant for biomedical applications. For biomedical decision-support applications, inherently interpretable models are often desirable because the decision process is more transparent and auditable than that of black-box models relying primarily on post hoc explanations [34]. In this study, ITFS-RM does not replace supervised classifiers; rather, it provides an additional explanatory layer. The classifiers evaluate whether the proposed features can distinguish HC from PD, whereas ITFS-RM further indicates which combinations of regional information-theoretic features form separable HC–PD rule spaces.

### 4.6. Relation to Deep Learning, Prior Insole Studies, and Wearable Biomedical Sensing

The comparison with feature-level deep learning and raw-signal baselines suggests that the proposed information-theoretic representation provides a compact and discriminative input space under the current experimental setting. Direct learning from extracted feature sequences or plantar insole sensor signals did not outperform the proposed information-theoretic features combined with conventional classifiers. One possible explanation is that the proposed features explicitly encode regional plantar loading properties, whereas deep learning models may require larger and more diverse datasets to learn similarly informative representations directly from raw or weakly structured inputs.

At the subject-independent subject-aggregated level, GradientBoosting with the proposed information-theoretic features achieved an accuracy of 0.8655 and an F1-score of 0.8723. In comparison, the best feature-level deep learning baseline under the same subject-independent subject-aggregated setting was MLP, with an accuracy of 0.7576 and an F1-score of 0.8095. These results suggest that compact information-theoretic features may be advantageous in small-sample subject-independent evaluation, where deep learning models may not have sufficient data diversity to learn stable representations.

Compared with representative insole sensor-based PD classification studies, the proposed method achieved competitive performance while retaining stronger interpretability. Existing studies have used frequency-domain features, time-domain features, raw time-series signals, spectrogram representations, multidomain features, and deep neural models. These methods can provide effective classification, but they often offer limited insight into which plantar regions or signal properties drive the classification. In contrast, the proposed framework links classification performance to regional plantar anatomy, information-theoretic features, ablation-based contribution analysis, and explicit ITFS-RM rule spaces. Therefore, the advantage of the proposed method lies in combining predictive performance with interpretable plantar-pressure representation.

The comparison with previous studies should be interpreted with caution because different studies may use different sample definitions, feature sets, subject-splitting strategies, and validation protocols. In Table 19, the proposed method is reported at three distinct levels: subject-independent subject-aggregated evaluation, walking-recording level evaluation, and gait-cycle level evaluation. The subject-independent subject-aggregated accuracy of 86.55% provides the most conservative comparison for unseen-subject generalization, whereas the 93.44% walking-recording level result and the 96.68% gait-cycle level result characterize discriminative performance under their corresponding sample definitions. These results should not be combined into a single claim of overall superiority.

The present study also fits within a broader trend in wearable biomedical sensing, in which wearable devices are increasingly designed not only to collect physiological signals but also to support interpretable and task-specific signal analysis. Recent wearable ECG dry-electrode work has emphasized long-term and unobtrusive physiological monitoring [35], while portable triboelectric sensing has been explored for vascular-access monitoring in dialysis patients [36]. Although these applications differ from plantar-pressure gait assessment, they highlight the broader need for wearable systems that combine practical sensing with interpretable physiological signal features. In this context, the present work contributes an information-theoretic plantar-signal representation for wearable gait assessment rather than a new sensing device.

### 4.7. Computational Feasibility, Confounders, and Parameter Robustness

The proposed framework has potential for near-real-time or offline rapid analysis because most information-theoretic descriptors are computed from low-dimensional regional signals rather than from high-dimensional raw sensor streams. The computational cost analysis showed that the mean signal duration was 121.17 s, whereas the mean full computation pipeline required 61.05 s. The mean information-theoretic feature extraction time was 60.43 s, and ITFS-RM three-dimensional feature-space construction required only 0.346 ms. These results suggest that the current implementation is faster than the recording duration for retrospective analysis and that the final rule-based ITFS-RM inference step is computationally lightweight.

However, the present implementation was evaluated as an offline processing pipeline rather than as an embedded streaming system. Real-time deployment would require streaming gait-event detection, incremental feature updating, memory-efficient histogram estimation, and device-level validation on the target hardware. Therefore, the current results support computational feasibility for near-real-time analysis, but they do not yet establish real-time operation on a wearable embedded platform.

The clinical interpretation of the proposed features was evaluated using covariate-adjusted and association analyses. In the HC–PD group comparison, covariate adjustment was performed using age, sex, and walking speed, because these variables were available for both groups. Among the 25 region–descriptor feature groups, 13/25 remained significant after age adjustment, 13/25 after sex adjustment, and 12/25 after walking-speed adjustment. Stratified analyses showed that representative features retained HC–PD differences across age and sex subgroups. UPDRS and Hoehn–Yahr stage were analyzed within the PD group as clinical-severity indicators rather than as covariates in HC–PD comparisons. Spearman correlation analysis summarized the associations between representative plantar information-theoretic features and demographic, walking-speed, and clinical-severity variables. These results indicate that the main HC–PD feature differences were retained after adjustment for the available shared covariates, whereas clinical-severity variables were evaluated separately within the PD group.

Therefore, the proposed features should be interpreted as candidate digital gait features for characterizing plantar-pressure dynamics and PD-related gait differences. The public dataset does not provide complete control over medication state, disease-side dominance, detailed task conditions, or all clinical covariates for every recording. As a result, the present analysis supports discriminative association with PD status, whereas its clinical diagnostic value requires further validation in larger and independent cohorts.

The methodological sensitivity analyses provide additional support for the stability of the feature extraction procedure. The number of significant features was unchanged across normalization settings, and five-region and sensor-level representations retained similar proportions of significant features. Both 10 and 12 histogram bins retained 10/33 significant distributional features, and sample entropy computation remained below 0.001 s across the tested tolerance settings. These findings reduce, but do not eliminate, concerns that the results were driven by a single preprocessing or parameter choice.

### 4.8. Limitations and Future Directions

Several limitations should be acknowledged. First, this study used a publicly available dataset and did not collect new participant data or use a self-developed plantar insole sensor or independent acquisition system. Therefore, the analysis was constrained by the sensor layout, walking protocol, signal quality, and completeness of clinical annotations in the original dataset. Although the use of public data supports reproducibility, the generalizability of the proposed framework should be further validated using independent cohorts, different insole devices, and real-world walking conditions.

Second, the public dataset contains repeated walking recordings from some participants and includes heterogeneous walking conditions, walking speeds, and task states. This structure makes subject-related data leakage an important methodological concern when recording files are split without subject grouping. Therefore, subject-independent holdout testing, subject-grouped cross-validation, LOSO validation, and subject-balanced cycle aggregation were used to provide more conservative evaluation. Nevertheless, gait-cycle level and walking-recording level results should be interpreted as sample-level and complete-recording separability analyses rather than as direct clinical deployment estimates. Performance interpretation should place greater emphasis on subject-independent validation, confidence intervals, and consistency across validation schemes than on small numerical differences between individual metrics.

Third, this study focused on binary HC–PD classification, and important factors such as medication status, disease-side dominance, detailed disease duration, disease severity, and standardized task information could not be fully controlled. Therefore, the proposed region-specific information-theoretic features should be interpreted as candidate digital gait features for characterizing plantar-pressure dynamics and PD-related gait differences. Future studies should further evaluate the associations between these features and clinical scales, disease progression, and functional outcomes and should validate their stability under stricter clinical adjustment.

Finally, this study used plantar-pressure signals only. Combining plantar information-theoretic features with inertial sensors, video-based gait parameters, electromyography, or clinical scales may provide a more comprehensive representation of Parkinsonian gait impairment. Future work should also conduct external validation, prospective real-world data collection, real-time system implementation, and model calibration under real-world walking conditions.

## 5. Conclusions

Region-specific information-theoretic analysis of wearable plantar insole signals provides a compact representation for HC–PD gait separation. By reorganizing bilateral plantar-pressure recordings into anatomical regions and extracting SII, EN, NEG, SEN, and KL features, the proposed framework characterized plantar-pressure dynamics from the perspectives of self-information fluctuation, probabilistic uncertainty, non-Gaussian organization, temporal irregularity, and directional distributional discrepancy. The results indicate that PD-related plantar-pressure differences were spatially heterogeneous, with stable contributions from toe-related and non-Gaussian descriptors, suggesting that regional loading organization and push-off-related plantar information provide useful complementary evidence for Parkinsonian gait assessment.

The classification results should be interpreted according to the evaluation level. Gait-cycle level and walking-recording level analyses demonstrated strong local-cycle and complete-recording separability, which is meaningful because walking speed, task state, and recording condition can influence plantar-pressure organization. However, these results should not be considered equivalent to subject-level clinical diagnosis when repeated recordings from the same participant are available. Subject-wise evaluation provided more conservative evidence of unseen-subject generalization, with subject-independent holdout accuracy reaching 0.8475, subject-balanced 50-cycle holdout accuracy reaching 0.8655, and LOSO validation showing lower but clinically more relevant performance.

ITFS-RM further provided an interpretable feature-space analysis by identifying explicit three-dimensional rule regions involving toe-region organization, forefoot signal complexity, KL-based regional transition, and bilateral asymmetry. These rule regions complement conventional classifier-based evaluation by showing how regional information-theoretic descriptors jointly contribute to HC–PD separability.

Overall, the proposed framework shows potential as an interpretable information-theoretic representation of plantar-pressure dynamics for PD gait assessment. The analysis was based on a public dataset with repeated recordings and incomplete control of potential confounders, including age, sex, disease severity, walking speed, medication status, and recording conditions. Therefore, the results should be interpreted within the scope of the available dataset and validation settings.

Future work should focus on external validation, stronger subject-wise generalization, real-world walking data, real-time implementation, and multimodal integration with inertial, video-based, electromyographic, or clinical measurements.

## Figures and Tables

**Table 1:** Parameter-stability analysis of information-theoretic feature extraction.

Analysis Item	Default Setting	Compared Settings	Main Result
Normalization	Sensor-wise maximum normalization	No normalization; sensor-wise, foot-wise, and recording-wise normalization	10/53 significant features in all settings
Region partition	Five plantar regions	Five-region and sensor-level representations	18.87% and 18.60% significant features
Histogram bins	10 bins	10 and 12 bins	10/33 significant features in both settings
Sample entropy	m=2,r=0.2×SD	r/SD=0.15,0.20,0.25	Computation time below 0.001 s

**Table 2:** Computational cost analysis of information-theoretic feature extraction and ITFS-RM three-dimensional feature-space construction.

Analysis Item	Mean Time
Signal recording duration	121.17 s
Information-theoretic feature extraction	60.43 s
ITFS-RM three-dimensional feature-space construction	0.346 ms
Full computation pipeline	61.05 s

**Table 3:** Performance of ITFS-RM under subject-grouped evaluation.

Evaluation Scheme	Level	*N*	Coverage	Accuracy	Precision	Recall	F1	Macro F1	AUC
Independent test	walking-recording level	80	100.0%	0.8250	0.8448	0.9074	0.8750	0.7917	0.7853
Independent test	Subject-aggregated	50	100.0%	0.7400	0.7273	0.8571	0.7869	0.7268	0.7638
5-fold CV	walking-recording level	226	100.0%	0.7965	0.8314	0.8938	0.8614	0.7391	0.7236
5-fold CV	Subject-aggregated	115	100.0%	0.7565	0.7229	0.9231	0.8108	0.7347	0.7237
LOSO	walking-recording level	226	100.0%	0.7788	0.8161	0.8875	0.8503	0.7133	0.6974
LOSO	Subject-aggregated	115	100.0%	0.7304	0.6932	0.9385	0.7974	0.6974	0.7135

**Table 4:** Retained three-dimensional ITFS-RM feature spaces and stage-wise assignments in the independent test cohort.

Stage	Retained Three-Dimensional Feature Space	Walking-Recording Level Assignment			Subject-Aggregated Assignment		
		Assigned PD	Assigned HC	Remaining	Assigned PD Subjects	Assigned HC Subjects	Remaining Subjects
1	$S_{1}=\left(NEG_{T,r},\;EN_{FF,l},\;\Delta KL_{r}^{RF,MF}\right)$	51	15	14	26	11	13
2	$S_{2}=\left(SII_{T,r},\;SEN_{FF,l},\;\Delta KL_{l}^{RF,MF}\right)$	7	4	3	4	4	5
3	$S_{3}=\left(SII_{T,l},\;SEN_{T,l},\;KL_{T}^{l\to r}\right)$	0	1	2	0	1	4

**Table 5:** Explicit decision rules retained by the final ITFS-RM model and their coverage in the development walking-recording level and independent subject-aggregated cohort.

Stage	Target Class	Explicit Rule	Walking-Recording Level			Subject-Aggregated		
			Target Coverage	Errors	Purity	Target Coverage	Errors	Purity
1	HC	$NEG_{T,r}\geq 1.01484$ and $EN_{FF,l}\leq 2.62961$	30	2	0.938	9	3	0.7500
1	PD	$NEG_{T,r}\leq 1.06573$ and $\Delta KL_{r}^{RF,MF}\leq 1.33196$	129	22	0.854	21	6	0.7778
2	HC	$SEN_{FF,l}\geq 0.0583528$ and $\Delta KL_{l}^{RF,MF}\geq 0.657353$	10	1	0.909	3	1	0.7500
2	PD	$\Delta KL_{l}^{RF,MF}\in \left[-0.170383,\;0.513442\right]$	22	3	0.880	2	2	0.5000
3	HC	$SEN_{T,l}\in \left[0.0538727,\;0.057009\right]$	2	0	1.000	1	0	1.0000
3	PD	$KL_{T}^{l\to r}\geq 0.209862$	6	1	0.857	0	0	0

**Table 6:** Gait-cycle level classification performance of different machine learning models on the validation and test sets. Bold values indicate the best performance for each metric, and the bold model name indicates the overall best-performing classifier.

Model	Validation Set					Test Set				
	Acc.	Prec.	Recall	F1	AUC	Acc.	Prec.	Recall	F1	AUC
LogisticRegression	0.8152	0.9050	0.8199	0.8604	0.8786	0.8125	0.9042	0.8165	0.8581	0.8786
DecisionTree	0.8860	0.9148	0.9217	0.9182	0.8636	0.8888	0.9179	0.9224	0.9201	0.8677
AdaBoost	0.8456	0.8707	0.9132	0.8915	0.9010	0.8437	0.8710	0.9097	0.8899	0.9003
GradientBoosting	0.8891	0.8914	0.9567	0.9229	0.9464	0.8903	0.8947	0.9543	0.9236	0.9467
RandomForest	0.9434	0.9334	0.9891	0.9605	0.9872	0.9501	0.9411	**0.9902**	0.9650	**0.9890**
SVM	0.9485	**0.9735**	0.9517	0.9625	0.9858	0.9472	**0.9754**	0.9479	0.9614	0.9858
MLP	0.9602	0.9694	0.9734	0.9714	0.9842	0.9617	0.9709	0.9741	0.9725	0.9837
**KNN**	**0.9636**	0.9656	0.9826	**0.9740**	**0.9884**	**0.9668**	0.9677	0.9850	**0.9763**	0.9876

**Table 7:** Walking-recording level classification performance of different machine learning models on the validation and test sets. Bold values indicate the best performance for each metric, and the bold model name indicates the overall best-performing classifier.

Model	Validation Set					Test Set				
	Acc.	Prec.	Recall	F1	AUC	Acc.	Prec.	Recall	F1	AUC
LogisticRegression	0.8710	**0.8750**	0.9545	0.9130	0.8232	0.8033	0.9167	0.7857	0.8462	0.8835
DecisionTree	0.7419	0.7692	0.9091	0.8333	0.6212	0.8361	0.8636	0.9048	0.8837	0.7945
AdaBoost	0.8387	0.8400	0.9545	0.8936	0.8939	0.8361	0.8478	0.9286	0.8864	0.9073
GradientBoosting	**0.8710**	0.8462	**1.0000**	**0.9167**	0.8081	0.8689	0.8542	**0.9762**	0.9111	0.8860
RandomForest	0.8387	0.8148	**1.0000**	0.8980	**0.9217**	0.8525	0.8667	0.9286	0.8966	**0.9135**
SVM	0.8065	0.8333	0.9091	0.8696	0.9192	0.8361	0.8636	0.9048	0.8837	0.8972
**MLP**	**0.8710**	0.8462	**1.0000**	**0.9167**	0.8434	**0.9344**	**0.9524**	0.9524	**0.9524**	0.8947
KNN	0.8065	0.8077	0.9545	0.8750	0.8333	0.8361	0.8478	0.9286	0.8864	0.8603

**Table 8:** Five-fold cross-validation performance at gait cycle and walking-recording levels. Values are reported as mean with 95% confidence interval.

Level	Model	Acc.	Prec.	Recall	F1	AUC
Gait cycle	LogisticRegression	0.8123 (0.8102–0.8143)	0.9026 (0.9014–0.9038)	0.8179 (0.8142–0.8216)	0.8582 (0.8563–0.8600)	0.8768 (0.8746–0.8789)
Gait cycle	DecisionTree	0.8902 (0.8875–0.8930)	0.9187 (0.9181–0.9193)	0.9237 (0.9197–0.9276)	0.9212 (0.9190–0.9233)	0.8691 (0.8670–0.8712)
Gait cycle	AdaBoost	0.8442 (0.8395–0.8489)	0.8708 (0.8662–0.8753)	0.9108 (0.9075–0.9142)	0.8904 (0.8872–0.8935)	0.8964 (0.8937–0.8991)
Gait cycle	GradientBoosting	0.8885 (0.8862–0.8908)	0.8928 (0.8909–0.8947)	0.9539 (0.9526–0.9553)	0.9224 (0.9208–0.9239)	0.9462 (0.9448–0.9476)
Gait cycle	RandomForest	0.9474 (0.9463–0.9485)	0.9383 (0.9375–0.9392)	0.9892 (0.9883–0.9902)	0.9631 (0.9623–0.9639)	0.9887 (0.9879–0.9895)
Gait cycle	SVM	0.9485 (0.9467–0.9503)	0.9734 (0.9716–0.9752)	0.9519 (0.9506–0.9532)	0.9625 (0.9612–0.9638)	0.9861 (0.9854–0.9868)
Gait cycle	MLP	0.9636 (0.9611–0.9660)	0.9734 (0.9709–0.9759)	0.9741 (0.9727–0.9756)	0.9738 (0.9720–0.9755)	0.9856 (0.9839–0.9874)
Gait cycle	KNN	0.9658 (0.9644–0.9672)	0.9673 (0.9654–0.9693)	0.9840 (0.9834–0.9847)	0.9756 (0.9746–0.9766)	0.9876 (0.9860–0.9892)
Walking recording	LogisticRegression	0.7975 (0.7235–0.8715)	0.8722 (0.8260–0.9185)	0.8317 (0.7650–0.8985)	0.8511 (0.7965–0.9056)	0.8537 (0.7986–0.9088)
Walking recording	DecisionTree	0.7616 (0.6900–0.8332)	0.8254 (0.7609–0.8899)	0.8417 (0.8047–0.8787)	0.8328 (0.7856–0.8799)	0.7100 (0.6086–0.8113)
Walking recording	AdaBoost	0.8334 (0.7937–0.8731)	0.8709 (0.8161–0.9257)	0.9011 (0.8532–0.9490)	0.8835 (0.8571–0.9098)	0.8816 (0.8455–0.9176)
Walking recording	GradientBoosting	0.8236 (0.7950–0.8523)	0.8464 (0.8191–0.8737)	0.9157 (0.8877–0.9437)	0.8791 (0.8612–0.8971)	0.8964 (0.8570–0.9359)
Walking recording	RandomForest	0.8367 (0.8173–0.8561)	0.8488 (0.8248–0.8728)	0.9344 (0.9245–0.9443)	0.8892 (0.8794–0.8991)	0.9150 (0.8831–0.9468)
Walking recording	SVM	0.8497 (0.7966–0.9028)	0.8903 (0.8470–0.9336)	0.8971 (0.8548–0.9394)	0.8932 (0.8556–0.9308)	0.9112 (0.8730–0.9493)
Walking recording	MLP	0.8399 (0.8003–0.8795)	0.8743 (0.8387–0.9099)	0.9019 (0.8796–0.9242)	0.8877 (0.8608–0.9145)	0.8935 (0.8494–0.9376)
Walking recording	KNN	0.8334 (0.7924–0.8744)	0.8568 (0.8300–0.8835)	0.9162 (0.8652–0.9671)	0.8846 (0.8544–0.9148)	0.8633 (0.7946–0.9321)

**Table 9:** Regional ablation analysis summarized by the rank-based ablation importance score across classifiers. A higher AIS indicates a stronger relative contribution of the corresponding plantar region.

Evaluation Metric	Heel	Rearfoot	Midfoot	Forefoot	Toe
Gait-cycle level Acc. AIS	50.00	15.62	59.38	25.00	100.00
Gait-cycle level F1 AIS	50.00	15.62	56.25	28.12	100.00
Walking-recording level Acc. AIS	35.94	57.81	48.44	42.19	65.62
Walking-recording level F1 AIS	37.50	57.81	46.88	42.19	65.62

**Table 10:** Information-theoretic feature-type ablation analysis summarized by the rank-based ablation importance score across classifiers. A higher AIS indicates a stronger relative contribution of the corresponding feature type.

Evaluation Metric	SII	Entropy	Negentropy	Sample Entropy	KL
Gait-cycle level Acc. AIS	3.12	95.31	79.69	40.62	31.25
Gait-cycle level F1 AIS	3.12	93.75	81.25	40.62	31.25
Walking-recording level Acc. AIS	51.56	39.06	54.69	57.81	46.88
Walking-recording level F1 AIS	54.69	39.06	57.81	51.56	46.88

**Table 11:** Cross-subject generalization performance on the subject-independent test set.

Classifier	Accuracy	Precision	Recall	F1	AUC
DecisionTree	0.6610	0.8889	0.5854	0.7059	0.6850
LogisticRegression	0.7627	0.8649	0.7805	0.8205	0.8103
KNN	0.7627	0.7872	0.9024	0.8409	0.6843
RandomForest	0.8136	0.8571	0.8780	0.8675	0.8862
GradientBoosting	0.8475	0.9000	0.8780	0.8889	0.8767
MLP	0.8475	0.8478	0.9512	0.8966	0.9119
SVM	0.8305	0.8444	0.9268	0.8837	0.8238
AdaBoost	0.8305	0.8974	0.8537	0.8750	0.8753

**Table 12:** Subject-grouped five-fold cross-validation performance.

Classifier	Accuracy	Precision	Recall	F1	AUC
DecisionTree	0.6990	0.8305	0.7149	0.7662	0.6855
LogisticRegression	0.6693	0.8463	0.6538	0.7315	0.7738
KNN	0.7664	0.8429	0.8214	0.8288	0.8016
RandomForest	0.7540	0.8652	0.7646	0.8106	0.8307
GradientBoosting	0.7540	0.8683	0.7656	0.8076	0.8295
MLP	0.7207	0.8333	0.7491	0.7871	0.7827
SVM	0.7638	0.8785	0.7687	0.8155	0.8381
AdaBoost	0.7660	0.8740	0.7762	0.8203	0.8212

**Table 13:** Pooled prediction performance under leave-one-subject-out validation.

Classifier	Accuracy	Precision	Recall	F1	AUC
DecisionTree	0.6078	0.8092	0.5748	0.6721	0.6345
LogisticRegression	0.7222	0.8686	0.7103	0.7815	0.8202
KNN	0.7614	0.8310	0.8271	0.8290	0.7679
RandomForest	0.8137	0.8985	0.8271	0.8613	0.8700
GradientBoosting	0.7451	0.8542	0.7664	0.8079	0.8035
MLP	0.7549	0.8492	0.7897	0.8184	0.8146
SVM	0.7288	0.8659	0.7243	0.7888	0.8258
AdaBoost	0.7614	0.8770	0.7664	0.8180	0.8351

**Table 14:** Combined ablation analysis of GradientBoosting on the subject-independent test set using accuracy as the evaluation metric.

Ablation Level	Removed Item	Baseline Accuracy	Accuracy After Removal	Accuracy Change
Information type	SII	0.8475	0.7627	−0.0848
	Entropy	0.8475	0.7966	−0.0509
	Negentropy	0.8475	0.7797	−0.0678
	Sample Entropy	0.8475	0.8136	−0.0339
	KL	0.8475	0.7627	−0.0848
Plantar region	Heel	0.8475	0.8136	−0.0339
	Rearfoot	0.8475	0.8305	−0.0170
	Midfoot	0.8475	0.7966	−0.0509
	Forefoot	0.8475	0.8136	−0.0339
	Toe	0.8475	0.8136	−0.0339

**Table 15:** Subject-balanced k-cycle holdout repeated evaluation results.

Classifier	Accuracy (95% CI)	Precision (95% CI)	Recall (95% CI)	F1 (95% CI)	AUC (95% CI)	Best Single-Run Accuracy
DecisionTree	0.7224 [0.7154, 0.7295]	0.6692 [0.6632, 0.6753]	0.9744 [0.9655, 0.9834]	0.7930 [0.7883, 0.7978]	0.8511 [0.8413, 0.8610]	0.7879
LogisticRegression	0.7745 [0.7691, 0.7800]	0.7826 [0.7801, 0.7851]	0.8122 [0.8011, 0.8234]	0.7967 [0.7908, 0.8027]	0.8365 [0.8325, 0.8405]	0.8182
KNN	0.8036 [0.7982, 0.8091]	0.7872 [0.7808, 0.7936]	0.8789 [0.8692, 0.8886]	0.8299 [0.8250, 0.8348]	0.8810 [0.8760, 0.8860]	0.8485
RandomForest	0.8618 [0.8570, 0.8667]	0.9036 [0.8949, 0.9124]	0.8378 [0.8285, 0.8470]	0.8686 [0.8639, 0.8733]	0.9015 [0.8994, 0.9035]	0.9091
GradientBoosting	0.8655 [0.8606, 0.8703]	0.9054 [0.8980, 0.9128]	0.8422 [0.8357, 0.8487]	0.8723 [0.8678, 0.8768]	0.8875 [0.8839, 0.8910]	0.9091
MLP	0.7042 [0.6964, 0.7121]	0.6714 [0.6644, 0.6785]	0.9000 [0.8924, 0.9076]	0.7687 [0.7633, 0.7741]	0.8347 [0.8283, 0.8411]	0.7879
SVM	0.7303 [0.7249, 0.7357]	0.6865 [0.6823, 0.6908]	0.9311 [0.9245, 0.9378]	0.7902 [0.7860, 0.7944]	0.8264 [0.8219, 0.8309]	0.7576
AdaBoost	0.8212 [0.8153, 0.8272]	0.8169 [0.8108, 0.8231]	0.8678 [0.8558, 0.8798]	0.8408 [0.8349, 0.8467]	0.8992 [0.8929, 0.9055]	0.8788

**Table 16:** Grouped cross-validation and LOSO validation results of the subject-cycle model.

Validation Setting	Accuracy	Precision	Recall	F1	AUC
Subject-grouped five-fold cross-validation	0.7695 [0.7578, 0.7812]	0.7719 [0.7581, 0.7856]	0.8548 [0.8472, 0.8623]	0.8083 [0.7995, 0.8172]	0.8267 [0.8136, 0.8398]
LOSO cross-validation	0.7212	0.7474	0.7634	0.7553	0.8475

**Table 17:** Combined feature ablation analysis of the subject-cycle model using GradientBoosting.

Ablation Level	Removed Item	Baseline Accuracy	Accuracy After Removal	Accuracy Change
Information type	SII	0.8655	0.8485	−0.0170
	Entropy		0.8232	−0.0423
	Negentropy		0.6717	−0.1938
	Sample Entropy		0.8202	−0.0453
	KL		0.7576	−0.1079
Plantar region	Toe	0.8655	0.6667	−0.1988
	Forefoot		0.7798	−0.0857
	Midfoot		0.8364	−0.0291
	Rearfoot		0.8152	−0.0503
	Heel		0.8313	−0.0342

**Table 18:** Comparison of information-theoretic features, feature-level deep learning baselines, and plantar insole sensor signal baselines. The best accuracy at each evaluation level is highlighted in bold.

Evaluation Level	Input Representation	Model	Acc.	Prec.	Recall	F1	AUC
Gait cycle	Information-theoretic features	KNN	**0.9668**	0.9677	0.9850	0.9763	0.9876
	Feature-level deep learning	CNN	0.8221	0.8716	0.8722	0.8719	0.8715
		LSTM	0.8021	0.8750	0.8342	0.8541	0.8307
		RNN	0.7178	0.7995	0.7922	0.7958	0.7002
		BiLSTM	0.8205	0.8518	0.8976	0.8741	0.8585
		CNN–LSTM	0.8148	0.8606	0.8750	0.8677	0.8654
		CNN–BiLSTM	0.8303	0.8715	0.8862	0.8788	0.8724
Walking recording	Information-theoretic features	MLP	**0.9344**	0.9524	0.9524	0.9524	0.8947
	Feature-level deep learning	CNN	0.8689	0.9048	0.9048	0.9048	0.9211
		LSTM	0.5410	0.7059	0.5714	0.6316	0.5251
		RNN	0.8033	0.8947	0.8095	0.8500	0.8897
		BiLSTM	0.7377	0.8421	0.7619	0.8000	0.7469
		CNN–LSTM	0.7213	0.7660	0.8571	0.8090	0.6604
		CNN–BiLSTM	0.6885	0.7447	0.8333	0.7865	0.6278
	Plantar insole sensor signal	LogisticRegression	0.7869	0.9143	0.7619	0.8312	0.8647
		DecisionTree	0.6557	0.7692	0.7143	0.7407	0.6203
		AdaBoost	0.8033	0.8000	0.9524	0.8696	0.8534
		GradientBoosting	0.7869	0.7636	1.0000	0.8660	0.7682
		RandomForest	0.7541	0.7455	0.9762	0.8454	0.8114
		SVM	0.7541	0.8140	0.8333	0.8235	0.8008
		MLP	0.8033	0.8571	0.8571	0.8571	0.8170
		KNN	0.6721	0.7391	0.8095	0.7727	0.6266
		CNN	0.8197	0.9429	0.7857	0.8571	0.9511
		LSTM	0.6721	0.8438	0.6429	0.7297	0.7657
		RNN	0.7049	0.8158	0.7381	0.7750	0.7368
		BiLSTM	0.7869	0.8718	0.8095	0.8395	0.8246
		CNN–LSTM	0.7869	0.9394	0.7381	0.8267	0.8634
Subject-independent subject-aggregated	Information-theoretic features	GradientBoosting	**0.8655**	0.9054	0.8422	**0.8723**	**0.8875**
	Feature-level deep learning	MLP	0.7576	0.7083	0.9444	0.8095	0.6815
		CNN	0.6061	0.6190	0.7222	0.6667	0.6370
		LSTM	0.5152	0.5556	0.5556	0.5556	0.6111
		RNN	0.6970	0.6667	0.8889	0.7619	0.6556
		BiLSTM	0.4242	0.4762	0.5556	0.5128	0.4259
		CNN–LSTM	0.6364	0.6875	0.6111	0.6471	0.6593
		CNN–BiLSTM	0.6061	0.6923	0.5000	0.5806	0.5963

**Table 19:** Accuracy comparison with representative insole sensor-based PD classification studies using the same public PD gait dataset. Reported accuracies are summarized according to the feature domain, evaluation setting, and classifier model described in each study.

Study	Domain Feature	Accuracy (%)	Classifier Model
[23]	Frequency-domain features	77.33	NN
[24]	Frequency-domain features	84.00	SVM
Proposed	Region-specific information-theoretic features (subject-independent subject-aggregated evaluation)	86.55	GradientBoosting
[25]	Time-domain and frequency-domain features	86.90	LDA
[26]	Raw time-series signals	87.97	HCT
[27]	Spectrogram image representations	88.17	CNN
[28]	Time-domain features	88.70	CNN
[29]	Raw time-series signals	89.92	LSTM-PSOGO
[30]	Time-domain features	89.97	BPANN
[22]	Multidomain features	90.22	AdaBoost
[31]	Time-domain and frequency-domain features	91.20	SVM
[25]	Time-domain features	91.58	LDA
Proposed	Region-specific information-theoretic features (walking-recording level evaluation)	93.44	MLP
[32]	Raw time-series signals	93.75	RNN
[21]	Three-domain features	94.44	MLP
Proposed	Region-specific information-theoretic features (gait-cycle level evaluation)	96.68	KNN

## Data Availability

The data presented in this study are available in PhysioNet at https://physionet.org/content/gaitpdb/1.0.0/. These data were derived from the following resource available in the public domain: Hausdorff, J.M.; et al. Gait in Parkinson’s Disease [20], available online at https://physionet.org/content/gaitpdb/1.0.0/ (accessed on 5 July 2026).

## Supplementary Materials

The following supporting information can be downloaded at https://www.mdpi.com/article/10.3390/bios16070391/s1, Table S1: File-level dataset audit and subject-level data partition. This supplementary table documents the walking-recording files used in this study and their corresponding subject-level data partition. The table accounts for 166 participants listed in the demographic records, including 306 available walking-recording files.
